# Identification of WRKY transcription factors involved in regulating the biosynthesis of the anti-cancer drug camptothecin in *Ophiorrhiza pumila*

**DOI:** 10.1093/hr/uhac099

**Published:** 2022-04-22

**Authors:** Can Wang, Xiaolong Hao, Yao Wang, Itay Maoz, Wei Zhou, Zhigang Zhou, Guoyin Kai

**Affiliations:** Laboratory for Core Technology of TCM Quality Improvement and Transformation, The Third Affiliated Hospital, School of Pharmaceutical Sciences, Zhejiang Chinese Medical University, Hangzhou, Zhejiang, 310053, China; Key Laboratory of Exploration and Utilization of Aquatic Genetic Resources Conferred by Ministry of Education, Shanghai Ocean University, Shanghai, 201306, China; Laboratory for Core Technology of TCM Quality Improvement and Transformation, The Third Affiliated Hospital, School of Pharmaceutical Sciences, Zhejiang Chinese Medical University, Hangzhou, Zhejiang, 310053, China; Laboratory for Core Technology of TCM Quality Improvement and Transformation, The Third Affiliated Hospital, School of Pharmaceutical Sciences, Zhejiang Chinese Medical University, Hangzhou, Zhejiang, 310053, China; Department of Postharvest Science, ARO, The Volcani Center, HaMaccabim Rd 68, POB 15159, Rishon LeZion, 7528809, Israel; Laboratory for Core Technology of TCM Quality Improvement and Transformation, The Third Affiliated Hospital, School of Pharmaceutical Sciences, Zhejiang Chinese Medical University, Hangzhou, Zhejiang, 310053, China; Key Laboratory of Exploration and Utilization of Aquatic Genetic Resources Conferred by Ministry of Education, Shanghai Ocean University, Shanghai, 201306, China; Laboratory for Core Technology of TCM Quality Improvement and Transformation, The Third Affiliated Hospital, School of Pharmaceutical Sciences, Zhejiang Chinese Medical University, Hangzhou, Zhejiang, 310053, China

## Abstract

Camptothecin is a chemotherapeutic drug widely used to treat various cancers. *Ophiorrhiza pumila* is an ideal plant model for the study of camptothecin production, with various advantages for studying camptothecin biosynthesis and regulation. The DNA-binding WRKY transcription factors have a key regulatory role in secondary metabolite biosynthesis in plants. However, little is currently known about their involvement in camptothecin biosynthesis in *O. pumila*. We identified 46 *OpWRKY* genes unevenly distributed on the 11 chromosomes of *O. pumila*. Phylogenetic and multiple sequence alignment analyses divided the OpWRKY proteins into three subfamilies. Based on spatial expression and co-expression, we targeted the candidate gene *OpWRKY6*. Overexpression of *OpWRKY6* significantly reduced the accumulation of camptothecin compared with the control. Conversely, camptothecin accumulation increased in *OpWRKY6* knockout lines. Further biochemical assays showed that *OpWRKY6* negatively regulates camptothecin biosynthesis from both the iridoid and shikimate pathways by directly downregulating the gene expression of *OpGES*, *Op10HGO*, *Op7DLH*, and *OpTDC*. Our data provide direct evidence for the involvement of WRKYs in the regulation of camptothecin biosynthesis and offer valuable information for enriching the production of camptothecin in plant systems.

## Introduction

As the leading cause of death globally, cancer can cause nearly 10 million deaths in a year, posing a severe threat to human health [1]. Treatment options for cancer include surgery, anti-cancer medicines, and radiation therapy, administered alone or in combination [2]. Camptothecin exhibits remarkable antitumor activity by inhibiting topoisomerase I enzyme, and its two derivatives (topotecan and irinotecan) are widely used as anti-cancer chemotherapeutic drugs [2, 3]. As a naturally occurring plant monoterpene indole alkaloid (MIA), camptothecin accumulates in various distantly related plants, including *Camptotheca acuminata* (Nyssaceae), *Nothapodytes nimmoniana* (Icacinaceae), and *Ophiorrhiza pumila* (Rubiaceae) [4–6]. Compared with woody camptothecin-producing plants, *O. pumila* has the advantages of a short growth cycle, high camptothecin content, and easy genetic transformation, which render it an ideal model plant for the study of camptothecin biosynthesis and regulation [1, 7].

**Figure 1:**
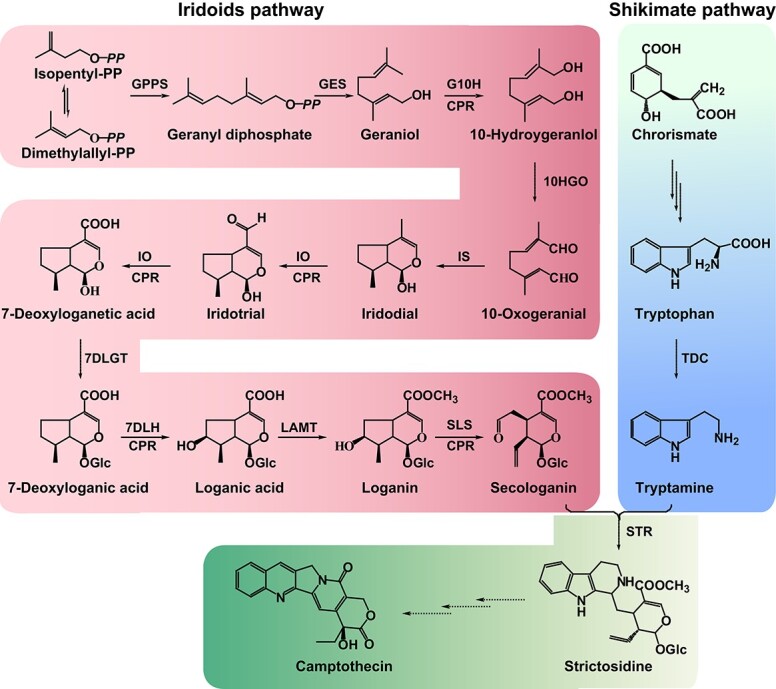
The putative camptothecin biosynthetic pathway in *O. pumila*. The three dashed arrows indicate that the camptothecin biosynthetic pathway in camptothecin-producing plants is still unknown.

Similar to other MIAs, camptothecin in *O. pumila* is synthesized from the important precursor strictosidine supplied by tryptamine in the shikimate pathway and secologain in the iridoid pathway (Fig. 1) [6, 8, 9]. In the shikimate pathway, tryptophan decarboxylase (OpTDC) catalyzes tryptamine production from tryptophan [10]. The iridoid pathway includes nine consecutive enzymatic steps initiated by geranyl diphosphate synthase (GPPS) and ending with the reduction of loganin to secologanin catalyzed by secologanin synthase (OpSLS) [11–13]. Strictosidine synthase (OpSTR) then catalyzes the formation of strictosidine from tryptamine and secologanin. Finally, strictosidine is converted to camptothecin through various biochemical reactions such as oxidation, reduction, cyclization, and isomerization [14]. Interestingly, the biosynthesis of strictosidine in *O. pumila* is distinct from that in *C. acuminata* [9, 15], in which strictosidinic acid serves as the final precursor. Still, the transcriptional regulation of camptothecin biosynthesis remains largely unknown in the camptothecin-producing plant *O. pumila*.

As a large family of transcription factors (TFs), the WRKYs have been reported to participate in various plant developmental and physiological processes and responses [16, 17]. The name WRKY refers to a highly conserved domain with nearly 60 amino acids that contains the signature WRKYGQK sequence and a non-typical zinc-finger-like motif (C_2_H_2_ or C_2_HC) [18, 19]. Several variants of the WRKYGQK motif have been reported, including WRKYGEK and WRKYGKK. Also, atypical WRKY motifs have been suggested, containing the consensus sequence W(R/K)(K/R)Y [20]. WRKY proteins were initially classified into three groups based on the number of WRKY domains and the type of zinc finger [18]. WRKY group I proteins contain two WRKYGQK motifs and a C_2_H_2_ zinc finger, whereas group III proteins contain one WRKYGQK motif and a C_2_HC zinc finger [19, 21]. Group II proteins contain one WRKY domain and a C_2_H_2_-type zinc finger, and they can be further classified into five subgroups according to the presence of short conserved structural motifs [22]. Recently, WRKY proteins have been found to regulate the biosynthesis of plant secondary metabolites, including various alkaloids. Their mode of action lies in their ability to bind the W-box in the promoters of their target genes [23–25]. For example, *HpWRKY44* was reported to regulate betalain biosynthesis in *Hylocereus polyrhizus* fruit by transcriptionally activating cytochrome P450-like (*HpCytP4501*) expression [26]. *CjWRKY1* acts as a transcriptional activator and positively regulates the biosynthesis of benzylisoquinoline alkaloids [27]. Both *CrWRKY1* in *Catharanthus roseus* and *OpWRKY2* in *O. pumila* positively regulate monoterpenoid indole alkaloid biosynthesis by directly affecting the expression of the *TDC* biosynthetic gene [7, 16]. *OpWRKY1* was shown to act as a negative regulator of camptothecin production in *O. pumila* hairy roots. Developmental changes in *O. pumila* hairy roots caused by overexpression of *OpWRKY3* resulted in increased accumulation of camptothecin, also promoting the transcription of *OpCPR* [28, 29]. In general, the WRKY transcription factors play an essential role in regulating the biosynthesis of specialized metabolites.

**Figure 2:**
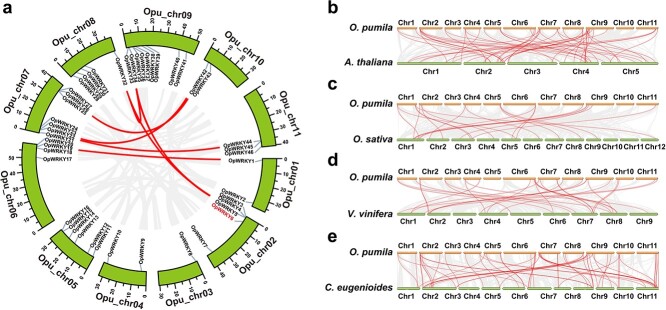
Chromosome-level analyses of the *OpWRKY* gene family in the *O. pumila* genome assembly. a. Chromosomal locations of *OpWRKY* genes and their synteny are illustrated by the circos diagram. Colored lines indicate similarity. b–e. Synteny analysis of *WRKY* genes between *O. pumila* and *A. thaliana*, *O. sativa*, *V. vinifera* and *C. eugenioides*, respectively.

With the mining of large-scale genome data, genome-wide identification and characterization of the WRKY transcription factors have been performed in a variety of plants, including *Arabidopsis thaliana* [21], *Oryza sativa* [30], *Glycine max* [31], *Populus trichocarpa* [32], *Vitis vinifera* [33], *Pyrus bretschneideri* [34], *Moringa oleifera* [35], and *Santalum album* [36]. However, there is little information on the identification of WRKY transcription factors and the functional characterization of camptothecin biosynthesis regulation in camptothecin-producing medicinal plants. The recent high-quality genome assembly of *O. pumila* provides an opportunity for genome-wide identification, characterization, and investigation of physiological functions of *WRKY* genes [1].

Here, forty-six *OpWRKY* genes were identified in the *O. pumila* genome, and their chromosomal locations, collinearity, classification, evolution, and expression patterns were analyzed. Based on our biochemical assays, OpWRKY6 was characterized as a negative regulator of *OpGES*, *Op10HGO*, *Op7DLH*, and *OpTDC* genes. OpWRKY6 binds to their promoters and simultaneously regulates the iridoid and shikimate pathways to reduce camptothecin production. Our identification and characterization of WRKY TFs in *O. pumila* and the functional characterization of OpWRKY6 as a regulator of camptothecin production provide novel insights and tools for re-engineering medicinal species enriched in valuable natural products.

## Results

### Identification and characterization of *WRKY* genes in the *O. pumila* genome

To identify candidate *WRKY* genes, an HMM search was performed against the *O. pumila* genome using the WRKY domain (PF03106), and 46 *OpWRKY* genes were obtained. *OpWRKY1–3* were reported previously, and *OpWRKY4*–*46* were named based on their positions on the *O. pumila* genome sequence (Table S1). Using the online CDD and SMART programs, the integrity of the WRKY domain was confirmed. Further characterization included analysis of subcellular localizations, amino acid lengths, molecular weights (MWs), and isoelectric points (pIs) (Table S1). Subcellular localization predictions indicated that all 46 proteins were found in the nucleus. Protein sequence lengths ranged from 195 (OpWRKY46) to 785 (OpWRKY41) amino acids, corresponding to MWs between 21.84 kDa (OpWRKY46) and 86.03 kDa (OpWRKY41), with an average of 44.08 kDa. The pIs varied from 4.77 (OpWRKY32) to 9.66 (OpWRKY33). All the data suggested a high variability among *WRKY* genes in the *O. pumila* genome. In addition, the number of WRKY family members in this study was much higher than that of WRKY members annotated in a previous transcriptome sequencing (Table S1 and S2). The present study provides a more comprehensive analysis of candidate *OpWRKY* genes.

As illustrated in Fig. 2a, 46 *OpWRKY* genes were disproportionately distributed across the 11 chromosomes of *O. pumila*. For example, *OpWRKY* genes were most abundant on Chr 9, with 9 genes, and least abundant on Chr 3, with only one gene (Fig. S1). Syntenic blocks within the *O. pumila* genome were examined to identify relationships among the *OpWRKY* genes and potential gene duplication events (Fig. 2a). Seven *OpWRKY* gene pairs were found in the *O. pumila* genome and were located on different chromosomes, indicating that segmental duplications in these regions probably contributed to expansion of the *OpWRKY* family.

**Figure 3:**
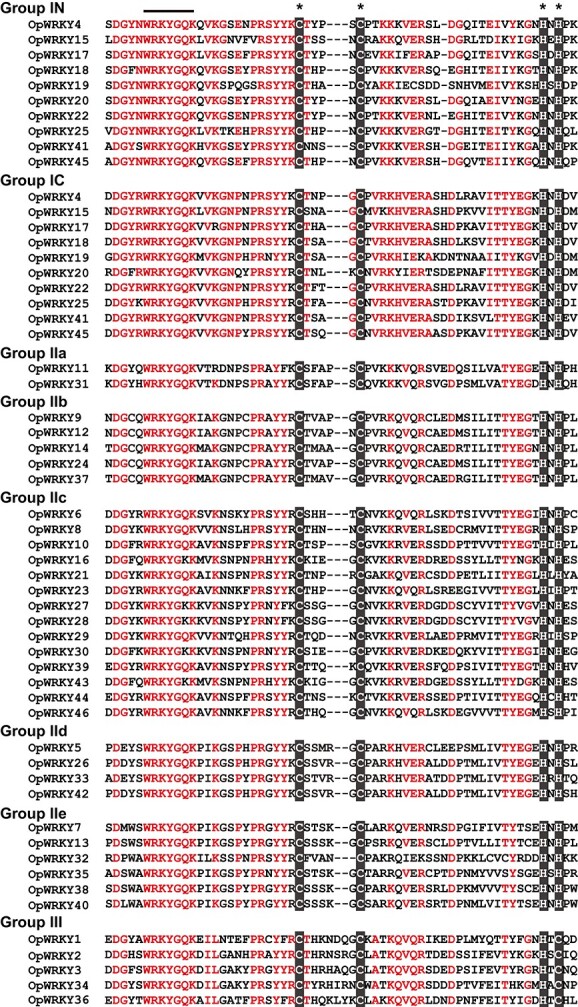
Protein sequence alignment of all OpWRKYs in *O. pumila*. The conserved WRKY domains are underlined, and the conserved zinc finger domains are highlighted in black boxes with asterisks.

Four comparative syntenic maps of *O. pumila* were constructed at the genome-wide level with four representative species, including one monocot (*O. sativa*) and three dicots (*Arabidopsis*, *V. vinifera*, and *Coffea eugenioides*) (Fig. 2b–e). A total of 40 *OpWRKY* genes showed syntenic relationships with those in *C. eugenioides*, followed by *Arabidopsis* (34), *V. vinifera* (18), and *O. sativa* (15) (Table S3). There were 53, 22, 19, and 63 orthologous pairs between the other four species (*Arabidopsis*, *O. sativa*, *V. vinifera*, and *C. eugenioides*). Collinear pairs were identified with the four other species (with 6 *OpWRKY* genes, *OpWRKY1*, *3*, *22*, *34*, *35*, and *44*), suggesting that these orthologous pairs may have existed before the ancestral divergence.

**Figure 4:**
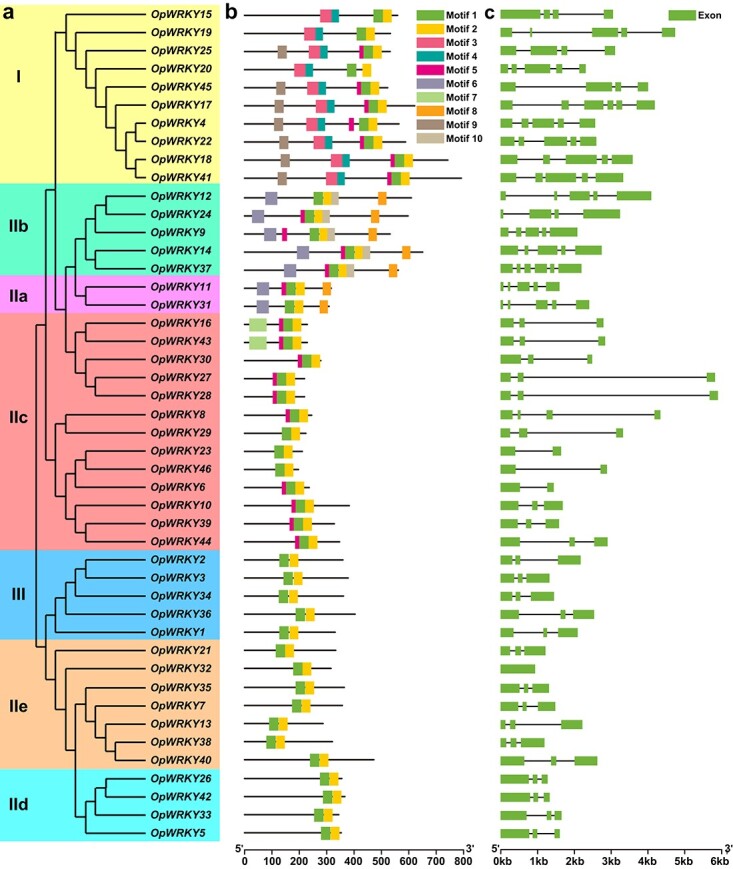
Gene structure and conserved motif analysis of *OpWRKY* genes. a. Phylogenetic analysis of OpWRKYs. b. Motif compositions of OpWRKY proteins. Ten motifs are shown in different colors, and their detailed information is provided in Table S5. c. Exon-intron structures of *OpWRKY* genes. Blue boxes and black lines represent exons and introns, respectively.

### Classification and phylogenetic relationships of *OpWRKY* genes

Multiple sequence alignments were performed to identify the structural features of the OpWRKY proteins. The majority of the 46 OpWRKY proteins (36/46, 78.3%) contained a single conserved WRKY domain, and the remaining 10 proteins (21.7%) contained two conserved WRKY domains (Fig. 3 and Table S4). In the WRKY domains, the WRKYGQK motif was relatively conserved across all OpWRKY family members, but five OpWRKYs differed at one residue, resulting in a WRKYGKK domain, similar to reports from other species such as tomato [37], *Sesamum indicum* [38], and *Cucumis sativus* [39]. All OpWRKY proteins had the C-X_4–7_-C-X_22–23_-H motif, forming C_2_HC or C_2_H_2_ zinc-finger structures.

The conserved WRKY domains from OpWRKYs and AtWRKYs were used to construct a phylogenetic tree and were classified into three groups (Fig. S2) [18]. Ten members belonged to Group I and contained two WRKY domains. Group II consisted of 31 OpWRKY proteins and was further divided into 5 subgroups, containing 2, 5, 14, 4, and 6 members in subgroups IIa–IIe, respectively. Five members of group III were found in the *O. pumila* genome, all of which contained a WRKYGQK domain and a C_2_HC-type zinc finger motif.

### Gene structure and conserved motif analysis of *OpWRKY* genes

Ten conserved motifs in the WRKY proteins of *O. pumila* were identified and are shown in Table S5. Motifs 1 and 2 were widely distributed in all OpWRKY proteins, corresponding to the conserved WRKY domain. Motifs 3, 4, and 9 were only found in the type I group. Motifs 6, 7, and 8, were only detected in group II; motifs 6 and 8 were only found in IIa and IIb; and motif 7 was only detected in IIc (Fig. 4b). The majority of proteins that belong to the same class based on similar motif compositions may have similar functions.

Exon-intron organization analysis of *OpWRKY* genes was also performed. The number of exons in the *OpWRKYs* ranged from one to six. Among the 46 *OpWRKY* genes, the majority (56.5%) were spliced with three exons and two introns (Fig. 4c). *OpWRKY* genes with similar structures clustered together, such as subgroup IId and group III with three exons and two introns or subgroup IIa with five exons and four introns. Group I and subgroup IIb contained 4–6 exons, subgroup IIc contained 2–4 exons, and subgroup IIe contained 3 exons, except for *OpWRKY32* (Fig. 4c). These results demonstrated that *OpWRKY*s exhibited diverse intron/exon structures.

In addition, *cis*-acting elements were analyzed in a 3000-bp regulatory region upstream of the ATG (promoter). Hormone-responsive (556), light-responsive (499), and stress-responsive (289) boxes were found in the promoter regions of *OpWRKY* genes (Table S6). Hormone-related elements for methyl jasmonate (MeJA) (186), abscisic acid (ABA) (155), ethylene (81), gibberellin (GA) (57), salicylic acid (SA) (50), and auxin (29) were also detected in the promoters of the *OpWRKY* genes. A number of stress-related promoter regions were identified in *OpWRKY* genes, including anaerobic induction (122), wound induction (57), drought (55), low-temperature (30), and defense (25) boxes. Meristem-related (49), circadian-related (19), and endosperm expression (13) *cis*-acting elements were also identified in the *OpWRKY* promoters. These results suggest that various *cis*-acting promoter elements may regulate *OpWRKY* genes during growth and stress responses.

### Co-expression network analysis of *OpWRKY* genes

Using the expression data, we performed hierarchical clustering and generated heat maps to visualize the expression of *OpWRKYs* and camptothecin biosynthesis genes in different tissues (Fig. S3). As illustrated in Fig. S3a, some *OpWRKY* genes had higher transcript accumulation in one or more tissues. For example, *OpWRKY3*, *OpWRKY6*, *OpWRKY9*, *OpWRKY10*, *OpWRKY14*, *OpWRKY20*, *OpWRKY23*, *OpWRKY24*, *OpWRKY38*, *OpWRKY40*, and *OpWRKY45* showed the highest expression levels in the root, consistent with most camptothecin biosynthesis genes (Fig. S3b). In addition, the expression levels of ten *OpWRKY* genes (*OpWRKY2*, *7*, *12*, *13*, *16*, *26*, *32*, *39*, *42*, and *43*) were higher in leaves than in stems and roots. These results indicated that *OpWRKY* genes have diverse tissue-specific expression patterns, suggesting a non-specific tissue-dependent role.

**Figure 5:**
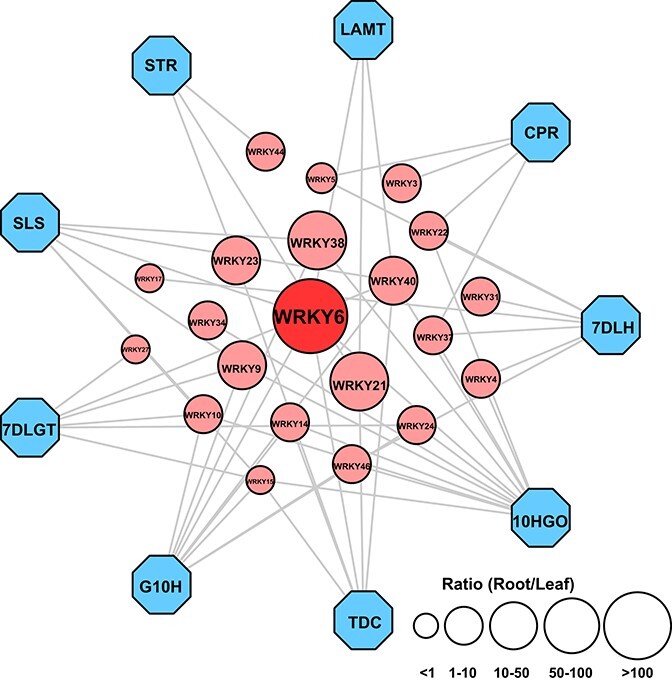
Correlation analysis of *OpWRKY* genes with camptothecin biosynthetic pathway genes in *O. pumila*. Blue octagons and red circles represent camptothecin biosynthetic pathway genes and *OpWRKY* genes, respectively. Larger circles indicate higher expression levels of *OpWRKY* genes in roots compared with leaves. Edges are drawn when the linear correlation coefficient is >0.8 with *p*-value <0.05.

A co-expression network was constructed between OpWRKYs and nine important camptothecin biosynthesis genes. As illustrated in Fig. 5, twenty *OpWRKYs* were strongly correlated with camptothecin biosynthesis genes. *OpWRKY6* and *OpWRKY40* were strongly positively correlated with 5 and 6 camptothecin biosynthesis genes, respectively. In addition, *OpWRKY15* was strongly negatively correlated with five camptothecin biosynthesis genes. Furthermore, the transcript level of *OpWRKY6* was much higher in roots than in leaves compared with other *OpWRKY* genes. Overall, this result showed that *OpWRKY6* may be involved in regulating camptothecin and its precursor biosynthesis.

Next, *OpWRKY6* with a 705-bp open reading frame (ORF) encoding 234 amino acids was isolated and functionally characterized. Protein sequence analyses showed that OpWRKY6 shared 68.84%, 58.10%, and 53.27% identity with PtWRKY43 (AZQ19338.1), PaWRKY19 (TKS06231.1), and TcWRKY56 (EOX95854.1), respectively. A phylogenetic tree was constructed, showing that OpWRKY6 clustered with AtWRKY43 in group IIc (Fig. S4) and contained a single conserved WRKY domain (Fig. S5). A subcellular localization assay of OpWRKY6-YFP fusion protein indicated that it was exclusively localized to nuclei in *Nicotiana benthamiana* leaf cells. By contrast, the control transformed with *pHB-YFP* showed fluorescence distributed throughout the cell (Fig. S6). These results demonstrated that OpWRKY6 regulates transcription as a TF in the nucleus.

### *OpWRKY6* negatively regulates camptothecin production in *O. pumila*

**Figure 6:**
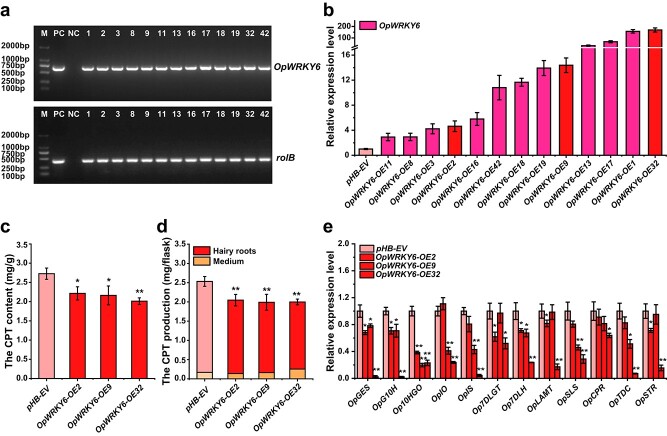
Overexpression of *OpWRKY6* in *O. pumila* hairy roots. a. Identification of positive *OpWRKY6*-*OE* hairy root lines. b. The expression levels of *OpWRKY6* in the *OpWRKY6*-*OE* lines. *OpUBQ* was used as the internal reference gene. c, d. The content (c) and production (d) of camptothecin in the *OpWRKY6*-*OE* lines. e. The transcript levels of camptothecin biosynthesis genes in the *OpWRKY6*-*OE* lines. All data represent the means ± SD of three biological replicates. Student’s t-test: ^*^p < 0.05; ^**^p < 0.01.

*OpWRKY6* overexpression (OE) transgenic hairy root lines were generated to examine the involvement of *OpWRKY6* in camptothecin biosynthesis regulation. Thirteen independent positive transgenic lines were verified by genomic PCR, and the positive rate for the *OpWRKY6*-*OE* lines was 52.4% (Fig. 6a). The *OpWRKY6* transcript levels were 2.9- to 169.6-fold higher in the *OpWRKY6*-*OE* lines than in the empty vector (EV) control lines, and three *OpWRKY6-OE* lines (OE-2, OE-9, and OE-32) with different higher expression levels were selected for further analysis (Fig. 6b). Compared with the control lines, *OpWRKY6*-*OE* transgenic hairy roots showed no significant differences in phenotype or biomass (Fig. S7). HPLC analysis indicated that the concentration of camptothecin in *OpWRKY6*-*OE* transgenic hairy roots was clearly decreased (Fig. 6c). Moreover, the camptothecin production of transgenic hairy roots and medium was significantly lower in the overexpression lines (Fig. 6d).

The expression levels of 12 camptothecin biosynthesis genes were analyzed in the transgenic lines. The camptothecin biosynthesis-related genes *Op10HGO*, *OpGES*, *OpG10H*, and *Op7DLH* showed a significant decrease in expression (qPCR analysis) in the *OpWRKY6*-*OE* transgenic lines (Fig. 6e). In addition, the transcript levels of *OpIO*, *OpSLS*, and *OpTDC* were lower in *OpWRKY6*-*OE9* and *OE32* transgenic lines. Notably, the transcript levels of 12 camptothecin biosynthesis-related genes were significantly lower in the *OpWRKY6*-*OE32* transgenic line compared with the other two lines. These qPCR results demonstrated that OpWRKY6 may control the biosynthesis of camptothecin, probably mainly by affecting the expression of *OpGES*, *Op10HGO*, *Op7DLH*, and *OpTDC*.

**Figure 7:**
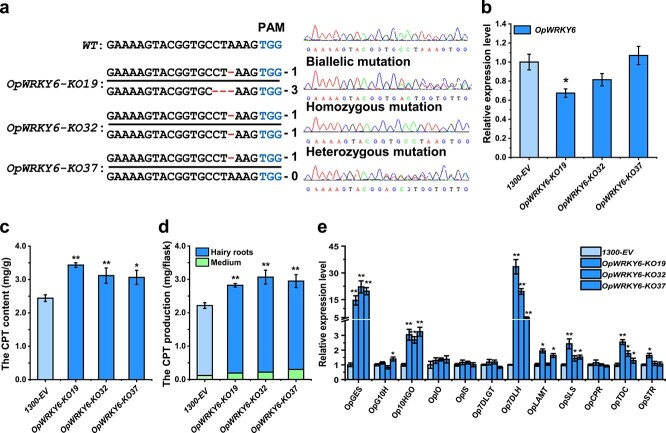
Knockout of *OpWRKY6* in *O. pumila* hairy roots. a. Genomic *OpWRKY6* DNA sequences from different *OpWRKY6-KO* lines were detected by DNA sequencing. b. The expression levels of *OpWRKY6* in the *OpWRKY6*-*KO* lines. c, d. The content (c) and production (d) of camptothecin in the *OpWRKY6*-*KO* lines. e. The expression levels of camptothecin biosynthesis genes in the *OpWRKY6*-*KO* lines. All data represent the means ± SD of three biological replicates. Student’s t-test: ^*^p < 0.05; ^**^p < 0.01.

To further elucidate the function of the OpWRKY6 TF in camptothecin biosynthesis, we attempted to knock out *OpWRKY6* of *O. pumila* using the CRISPR/Cas9 system. Sixty-nine independent lines were generated, with 18 positive lines (20.3% positive rate) (Fig. S8). Three independent lines were selected (KO-19, KO-32, and KO-37). The sequencing chromatograms suggested that the transgenic KO-32 line was homozygous, with mutations in both alleles at the same DNA locus (Fig. 7a). The KO-19 line was found to have biallelic mutations (two distinct variations), and the KO-37 line had a heterozygous mutation (wild-type/single mutation). The expression of *OpWRKY6* was significantly lower in the transgenic KO-19 line, slightly lower in the KO-32 line, and not significantly different from the control in the KO-37 line (Fig. 7b). The phenotypes and biomass of *OpWRKY6-KO* transgenic hairy roots were not significantly different from those of the wild-type control (Fig. S9). HPLC analysis indicated that the content and production of camptothecin were significantly higher in the *OpWRKY6-KO* lines compared with the control (Fig. 7c, d). The increased accumulation of camptothecin in the knockout lines was consistent with the upregulation of *OpGES*, *Op10HGO*, *Op7DLH*, *OpLAMT*, *OpSLS*, and *OpTDC* (Fig. 7e). These results demonstrated that OpWRKY6 was a TF that negatively regulated camptothecin biosynthesis.

### OpWRKY6 acts as a negative regulator of camptothecin biosynthesis genes

**Figure 8:**
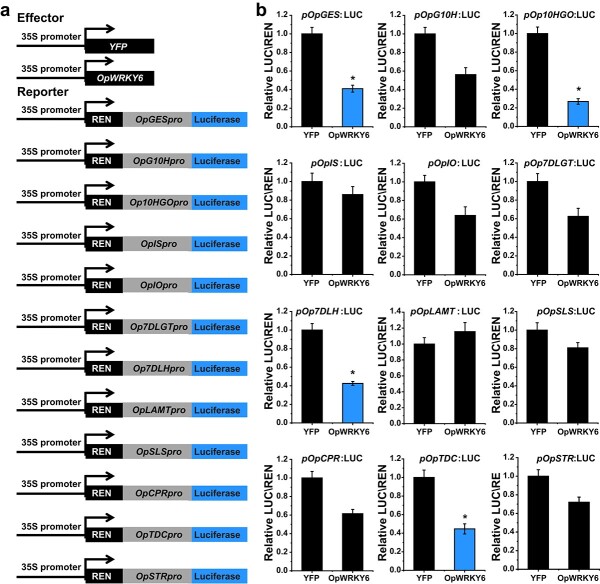
Dual-luciferase (Dual-LUC) assays showed the suppression effect of OpWRKY6 on the promoters of camptothecin biosynthesis genes. a. Schematic diagrams of the effector and reporter plasmids used in Dual-LUC assays. REN, *Renilla luciferase* internal reference gene. LUC, *firefly luciferase* reporter gene. b. Dual-LUC assay in *N. benthamiana* leaf cells using the constructs shown in (a). The empty vector *pHB*-YFP was used as the control. The relative LUC activity was normalized to the reference Renilla (REN) luciferase. Error bars indicate the SD (n = 3). Student’s t-test: ^*^p < 0.05.

Dual-luciferase assays were performed to verify that OpWRKY6 negatively regulates camptothecin biosynthesis genes. *proOpGES*, *proOpG10H*, *proOp10HGO*, *proOp7DLH*, *proOpLAMT*, *proOpSLS*, *proOpCPR*, *proOpTDC*, and *proOpSTR* promoter regions were used to drive the luciferase (LUC) gene as fusion reporters. Overexpression of *OpWRKY6* under the control of the 35S promoter was used as an effector (Fig. 8a). OpWRKY6 repressed the promoters of *OpGES*, *Op10HGO*, *Op7DLH*, and *OpTDC* compared with controls as measured by changes in LUC luminescence (Fig. 8b). These results showed that OpWRKY6 transcriptionally downregulates these four camptothecin biosynthesis genes.

**Figure 9:**
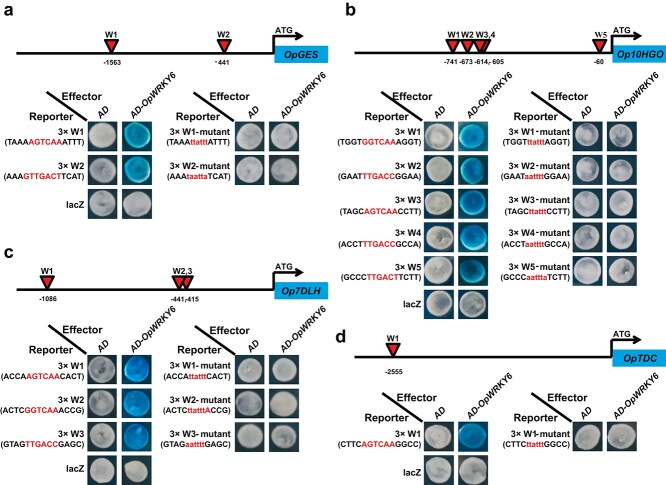
OpWRKY6 protein could directly bind to the W-box in the promoters of four camptothecin biosynthesis genes, as shown by yeast one-hybrid (Y1H) assays. a–d. Schematic diagrams of the *OpGES*, *Op10HGO*, *Op7DLH*, and *OpTDC* promoters. The positions of potential W-boxes are shown as red triangles. Y1H assays were repeated three times.

**Figure 10:**
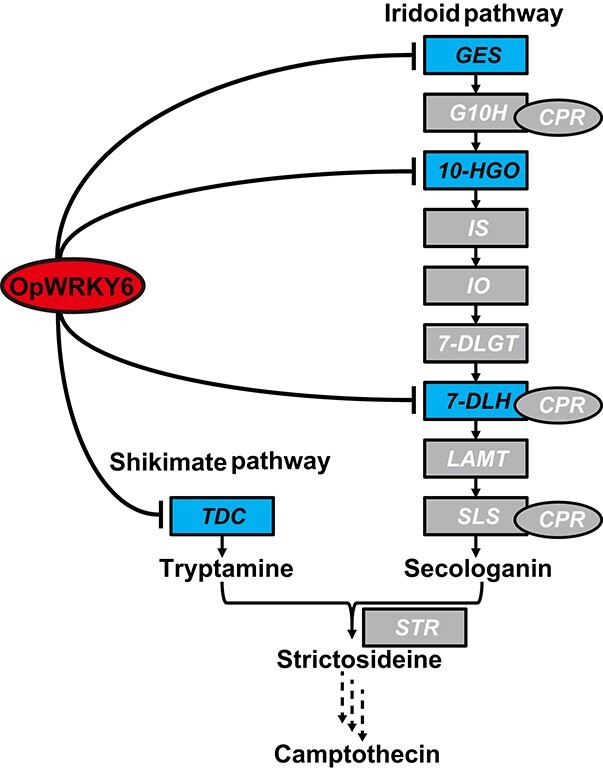
Model of the regulation of camptothecin biosynthesis by OpWRKY6 in *O. pumila*. The OpWRKY6 transcription factor negatively regulates camptothecin biosynthesis by directly binding and inhibiting biosynthesis genes in the shikimate pathway (*OpTDC*) and iridoid pathway (*OpGES*, *Op10HGO*, and *Op7DLH*).

The promoter sequences of camptothecin biosynthesis-related genes were analyzed to better understand the *OpWRKY6* mode of action. The *OpGES* promoter contained two W-box *cis*-elements, W1 and W2, that were 1563 and 441 bp upstream of the translation start site (ATG), respectively (Fig. 9a). The *Op10HGO* promoter contained five W-boxes, W1–W5, which were 741, 673, 614, 605, and 60 bp upstream of the ATG, respectively (Fig. 9b). Three W-boxes (W1, W2, and W3) were found in the promoter region of *Op7DLH* (Fig. 9c), whereas a single W-box was identified in the *OpTDC* promoter (Fig. 9d). In all cases, OpWRKY6 was able to bind the W-box in the promoter region, but not the mutant W-box, and the empty vector served as a negative control. Consequently, our results revealed that OpWRKY6 negatively regulates camptothecin biosynthesis by directly binding to W-boxes in the promoter regions of *OpGES*, *Op10HGO*, *Op7DLH*, and *OpTDC*, thereby repressing their expression (Fig. 10).

## Discussion

WRKY proteins are a class of transcriptional regulators found in higher plants [25]. To date, systematic and comprehensive genome-wide analyses of the WRKY family have been performed in a variety of plants [17]. However, systematic identification and functional characterization of WRKY proteins have rarely been performed in camptothecin-producing plants. The genome of *O. pumila* has been published, providing useful tools for genome-wide analysis of the *OpWRKY* gene family [1]. In the present study, we identified 46 *OpWRKY* genes based on the *O. pumila* genome sequence, similar to the 47 *WRKY* genes in *Ricinus communis* [40], 49 in *Coffea canephora* [41], and 45 in *Hordeum vulgare* [42]. By contrast, more WRKY members were reported in *Glycine max*, *Malus domestica*, *O. sativa*, *Arabidopsis*, and *V. vinifera* (Table S7). Out of the 46 *OpWRKY* genes, only 14 were found in the genome data and not in the transcriptome data of *O. pumila* roots and hairy roots (Table S1 and S2). This difference can be explained by low gene expression or by the presence of pseudogenes. Conversely, two *OpWRKY* genes (OpWRKY12 and 21) were found only in the transcriptome data [7], possibly caused by alternative splicing or incomplete genome assembly. In addition, only 34 WRKY genes were identified in a previous *O. pumila* genome using the blast_rbh.py script. Our comprehensive analysis of the *OpWRKY* gene family therefore provides more meaningful data for exploring the characteristics and functions of *WRKY* family members.

WRKYs can be classified into three groups based on the motif features of the zinc finger and the number of WRKY domains. Our data show that the 46 *OpWRKYs* are divided into three distinct groups, among which 31 *OpWRKYs* were assigned to group II, accounting for the largest proportion (67.4%). These results were similar to reports from *A. thaliana* [18], cucumber [39], and *C. canephora* [41]. In cucumber, 41 of 61 CsWRKY proteins were assigned to group II, and in *C. canephora*, 34 (69.39%) CcWRKY proteins belonged to group II. Moreover, variations in the conserved WRKYGKK motif were observed in OpWRKY16, OpWRKY27, OpWRKY28, OpWRKY30, and OpWRKY43 proteins from the WRKY IIc subgroup. Similar cases have been found in other plant species such as *C. sativus* (CsWRKY10 and CsWRKY47), *Ananas comosus* (AcWRKY14, AcWRKY23, AcWRKY27, and AcWRKY43), and *M. oleifera* (MoWRKY24) [35, 39, 43]. Because changes in the WRKYGQK pattern may influence normal interactions of OpWRKYs with target genes, the binding specificities and functions of these five OpWRKY proteins may warrant further study. In addition, *OpWRKY* genes with similar gene structures and motif compositions always clustered in the same class, such as groups IId and III that contained three exons and two introns (Fig. 4). These points supported the phylogenetic results based on motif compositions, conserved protein architecture, and similarity in gene structures.

WRKY TFs have been found to play important roles in plant development and defense response. In *A. thaliana*, AtWRKY41 is an important regulator of abscisic acid insensitive 3 (ABI3) expression, conferring reduced primary seed dormancy [44]. Three WRKY transcription factors (AtWRKY46, AtWRKY54, and AtWRKY70) have been reported to participate in BR-regulated plant growth by cooperating with BES1 to regulate BR signaling [45]. In this study, the tissue expression profiles of *OpWRKYs* showed that most *OpWRKY* genes had diverse tissue-specific expression patterns; for example, *OpWRKY6* was highly expressed in roots. In addition, hormone-responsive (556), light-responsive (499), and stress-responsive (289) boxes were found in the promoter regions of *OpWRKY* genes (Table S6). These findings revealed that the *OpWRKY* genes may be regulated by various *cis-*acting elements in their promoters during growth and stress responses.

Emerging experimental data demonstrate that WRKY transcription factors can regulate a variety of plant secondary metabolites, including terpenoids, phenylpropanoids, and alkaloids [25]. In *Artemisia annua*, AaWRKY9 was reported to positively regulate the expression of *AaDBR2* and *AaGSW1* and increase artemisinin accumulation [46]. *SlWRKY73* activates the expression of three monoterpene synthase genes in *Solanum lycopersicum* [47]. In *H. polyrhizus*, *HpWRKY44* transcriptionally activates HpCYP450-like1, which regulates betalain biosynthesis [26]. In *C. roseus*, CrWRKY1 was shown to regulate the monoterpenoid indole alkaloid pathway by activating the central pathway gene *CrTDC* [16]. In *O. pumila*, we reported the involvement of three Group III WRKY TFs in the regulation of camptothecin biosynthesis. Analysis of *OpWRKY3* transgenic hairy roots indicated that *OpWRKY3* regulated camptothecin production by affecting the development of hairy roots [28]. *OpWRKY1* negatively regulates camptothecin biosynthesis by binding to the *OpCPR* promoter, whereas *OpWRKY2* acts as a positive regulator of camptothecin biosynthesis by activating the expression of *OpTDC*. However, other WRKY subfamilies with important roles in camptothecin biosynthesis have not been reported.

We constructed a co-expression network between all *OpWRKYs* and nine key camptothecin biosynthesis genes. These results indicated that *OpWRKY6* showed strong transcriptional overlap with five camptothecin biosynthesis genes (Fig. 5). Multiple sequence alignment and phylogenetic tree analysis revealed that OpWRKY6 clustered with AtWRKY43 from group IIc. *OpWRKY6* expression was tightly negatively correlated with transcript levels of camptothecin biosynthesis genes and camptothecin production in *OpWRKY6-OE* and *OpWRKY6-KO* transgenic hairy root lines (Fig. 6 , 7). Moreover, based on *in vivo* and *in vitro* biochemical experiments, *OpWRKY6* can selectively bind to W-box promoter elements in four camptothecin biosynthesis genes (*OpGES*, *Op10HGO*, *Op7DLH*, and *OpTDC*). These enzymes may control the flow of iridoid and tryptamine metabolism in camptothecin biosynthesis. Taken together, our results show that the OpWRKY6 TF acts as a negative regulator of camptothecin biosynthesis in *O. pumila* by directly regulating biosynthesis genes in the iridoid and shikimate pathways (Fig. 10). Therefore, our results reveal the molecular mechanism by which OpWRKY6 regulates camptothecin biosynthesis and provide a strategy for increasing camptothecin content.

## Conclusion

In this work, we first provided a systemic analysis of *OpWRKY* genes and then identified 46 *OpWRKYs* based on the *O. pumila* genome sequence. We identified *OpWRKY6* using co-expression network analysis between all *OpWRKYs* and camptothecin biosynthesis genes. *OpWRKY6* serves as a negative regulator of camptothecin biosynthesis by directly binding to and inhibiting the promoters of *OpGES*, *Op10HGO*, *Op7DLH*, and *OpTDC*. This genome-wide diversity analysis of *WRKY* genes in *O. pumila* provides new insights for future functional characterization of the regulation of plant specialized metabolites such as monoterpenoid indole alkaloids.

## Materials and methods

### Identification of candidate *OpWRKY* genes in the *O. pumila* genome

The *O. pumila* genome was downloaded from the *O. pumila* Genome DataBase, and putative *OpWRKY* TF genes in *O. pumila* were identified using HMMER3.2 with the WRKY domain (Pfam, PF03106). Subsequently, two online programs, CDD and SMART, were used to manually confirm whether all candidate OpWRKY proteins contained conserved WRKY domains. The amino acid lengths, MWs, and pIs of all OpWRKY proteins were determined using the ExPASy website, and their subcellular localizations were predicted with PSORT software [48].

### Chromosomal locations of *OpWRKYs* and gene duplications in the *O. pumila* genome

The chromosomal distributions of *OpWRKY* genes were displayed using TBtools software. MCScanX and BLASTP were used to analyze gene duplication events of *OpWRKY* genes in the *O. pumila* genome [49]. The synonymous relationships between *OpWRKY* genes and *A. thaliana*, *O. sativa*, *C. eugenioides*, and *V. vinifera WRKY* genes were displayed with the TBtools program.

### Protein sequence alignment and phylogenetic analyses

Protein sequence alignments of all conserved WRKY domains were performed with Clustal software. WRKY domain regions of *O. pumila* and *A. thaliana* were aligned after retrieval from the TAIR database. A neighbor-joining phylogenetic tree was constructed using MEGA 7.0 with 1000 bootstrap replicates [50].

### Gene structure and motif composition analysis

The coding sequences (CDSs) of *OpWRKYs* and their corresponding genomic sequences were used to predict exon-intron structures. Exon-intron structures of *OpWRKY* genes were visualized using GSDS v2.0 software [51]. The conserved motifs of OpWRKY proteins were investigated using the MEME v5.1.1 online program [52]. A schematic diagram of gene structures and conserved motifs was displayed and re-edited with TBtools software.

### Promoter *cis*-acting element analysis

The 3000-bp sequences upstream of the translation start codons of the *OpWRKY* genes were submitted to the PlantCARE database [53] to analyze the promoter regions. The binding elements for *OpWRKY* genes were searched by analyzing the 3000-bp promoter sequences of 12 genes encoding key enzymes of the camptothecin biosynthetic pathway using the PlantPAN 3.0 database [54].

### Co-expression network of *OpWRKYs* and camptothecin biosynthesis genes

To obtain candidate *OpWRKYs* for the regulation of camptothecin biosynthesis, the tissue expression patterns of all *OpWRKYs* and camptothecin biosynthesis genes were used to construct a co-expression network using the PCC (Partial Correlation Coefficients) method in the R platform between each set of variables to calculate Pearson correlation coefficients [55]. The correlation network was displayed using Cytoscape software (version 3.7.2) [56].

### Subcellular localization assay

First, *Agrobacterium tumefaciens* GV3101 strains harboring the *pHB-YFP* or *pHB-OpWRKY6-YFP* plasmids were separately infiltrated into leaves of *N. benthamiana*. Then, the YFP signals were observed using a confocal laser microscope (Carl Zeiss, Germany) after two days of infiltration. Nuclei were stained with 20 μg/mL 4, 6-diamidino 2-phenylindole (DAPI, Sigma) before observation.

### Generation of OpWRKY6 transgenic *O. pumila* hairy roots

*OpWRKY6* overexpression lines of *O. pumila* hairy roots were generated using the recombinant *pHB-OpWRKY6-YFP* vector. An *OpWRKY6* knockout vector was constructed using the CRISPR/Cas9 system as reported previously [13]. The empty *pHB*-YFP and pCAMBIA1300 vectors were used as controls for overexpression and knockout of *OpWRKY6*, respectively. All plasmids were isolated and transferred into *A. tumefaciens* strain C58C1, which was then used to transform *O. pumila* stems to generate hairy roots as previously described (Fig. S10) [15, 28]. Positive transgenic lines (*OpWRKY6-OE* and *OpWRKY6-KO*) were verified by PCR using the relevant primers (Table S8). PCR products of *OpWRKY6-KO* lines were cloned into the pMD19T vector (Takara), and the plasmids of individual colonies were sequenced by the Sunya company (Hangzhou, China). Positive transgenic hairy roots of *OpWRKY6-OE* and *OpWRKY6-KO* were cultured in B5 liquid medium under dark conditions at 25°C and 120 rpm for 40 days.

### RNA extraction and qRT-PCR analysis

Samples of roots, stems, and leaves were collected from 2-month-old sterile *O. pumila* plants to determine the tissue expression patterns of all *OpWRKY* genes. Transgenic hairy roots were collected to analyze the expression of *OpWRKY6*, *OpTDC*, *OpGES*, *OpG10H*, *Op10HGO*, *Op7DLH*, *OpCPR*, *Op7DLGT*, *OpIO*, *OpIS*, *OpLAMT*, *OpSLS* and *OpSTR* in *OpWRKY6-*OE and *OpWRKY6-*KO lines. Total RNA was isolated from the three different tissues and *OpWRKY6*-*OE* and *OpWRKY6*-*KO* transgenic lines, and qRT-PCR was performed with three biological replicates as described previously [7]. All gene-specific primers used in this study are listed in Table S8. The relative gene expression levels in different samples were calculated relative to that of *OpUBQ* (Ubiquitin) using the 2^−ΔΔCt^ method.

### Determination of camptothecin in *O. pumila* hairy roots

Samples of different hairy root lines (*OpWRKY6-OE*, *OpWRKY6-KO*, and empty vector controls) were freeze-dried and ground into powder. Extraction of camptothecin from transgenic hairy root lines and their corresponding media were performed as described previously [7]. High-performance liquid chromatography (HPLC) was used to determine the concentration of camptothecin.

### Dual-luciferase assays

The recombinant *pHB-OpWRKY6-YFP* vector was used as an effector to determine the regulatory role of the OpWRKY6 TF in the camptothecin biosynthetic pathway. For the reporter constructs, the promoters of the *OpTDC*, *OpGES*, *OpG10H*, *OpCPR*, *Op10HGO*, *Op7DLH*, *Op7DLGT*, *OpIO*, *OpIS*, *OpLAMT*, *OpSLS*, and *OpSTR* biosynthesis genes were inserted into the *pGREEN0800-LUC* vector and separately transformed into *A. tumefaciens* GV3101 strains. The empty *pHB-YFP* vector was used as the control. Infiltration into leaves of *N. benthamiana* and detection of luciferase activities were carried out as described previously [7]. Three biological replicates were performed for each combination.

### Yeast one-hybrid assays

To explore the regulatory mechanism of *OpWRKY6* in camptothecin biosynthesis, yeast one-hybrid assays (Y1H) were carried out as described previously [57]. First, the ORF sequence of *OpWRKY6* was fused to the *pB42AD* effector vector. Three tandem copies of the W-box or a mutant-W-box from the promoter regions of camptothecin biosynthesis genes were separately inserted into the *pLacZ* reporter vector. Then, EGY48 yeast strains were prepared with different combinations of effector and reporter constructs and cultured on selective medium (SD/−Ura/−Trp/+X-gal) for 2 days.

## Supplementary Material

Web_Material_uhac099

## Data Availability

The data generated or analyzed in the present research are included in this published article and its supplemental data files and available from the corresponding author upon reasonable request.
