# Supplementary material for: Identification of WRKY transcription factors involved in regulating the biosynthesis of the anti-cancer drug camptothecin in Ophiorrhiza pumila
Source: Hortic Res. 2022 Apr 22;9:uhac099. doi: 10.1093/hr/uhac099 (PMC9250654; doi:10.1093/hr/uhac099)
Supplement: Web_Material_uhac099 [file web_material_uhac099.docx]

Supplementary **information**


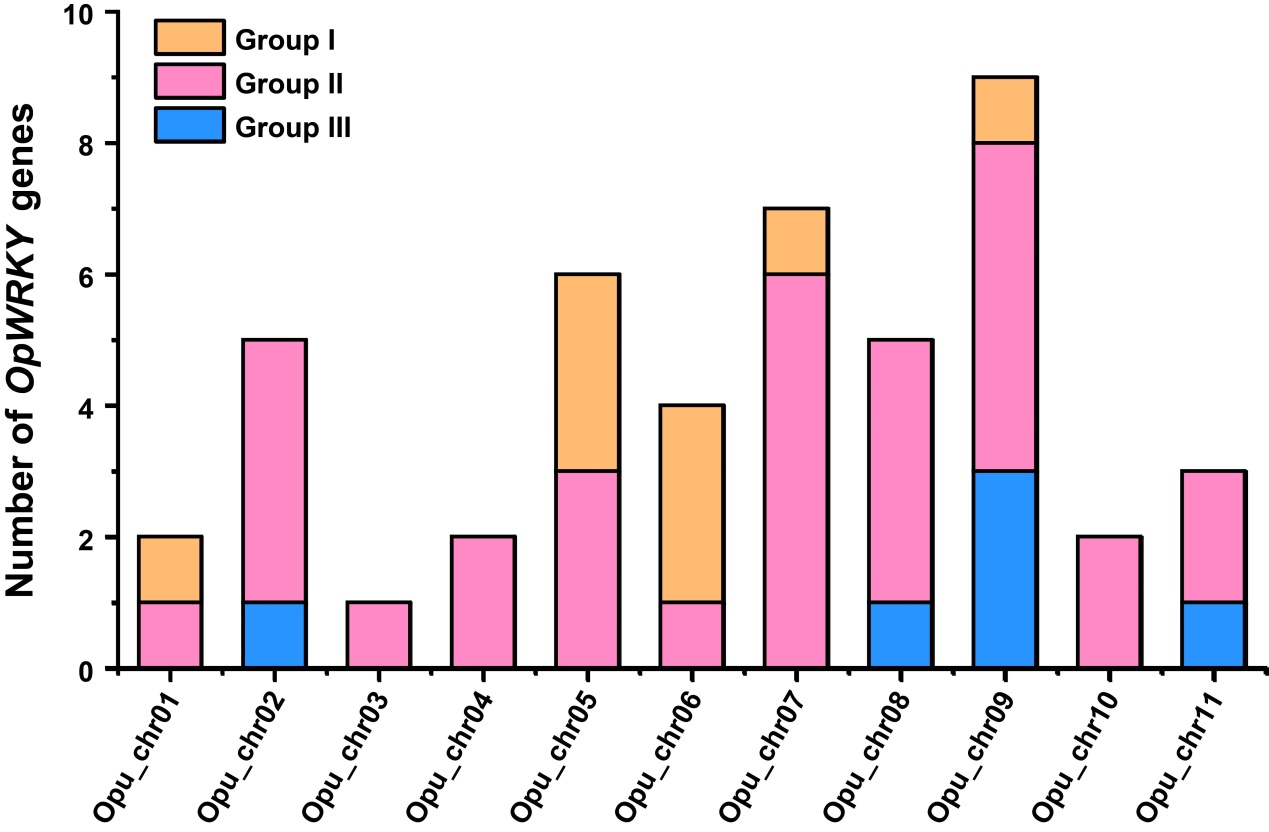


Supplementary **Figure 1.** The number of *WRKY* subfamily members on the *O. pumila* chromosome.


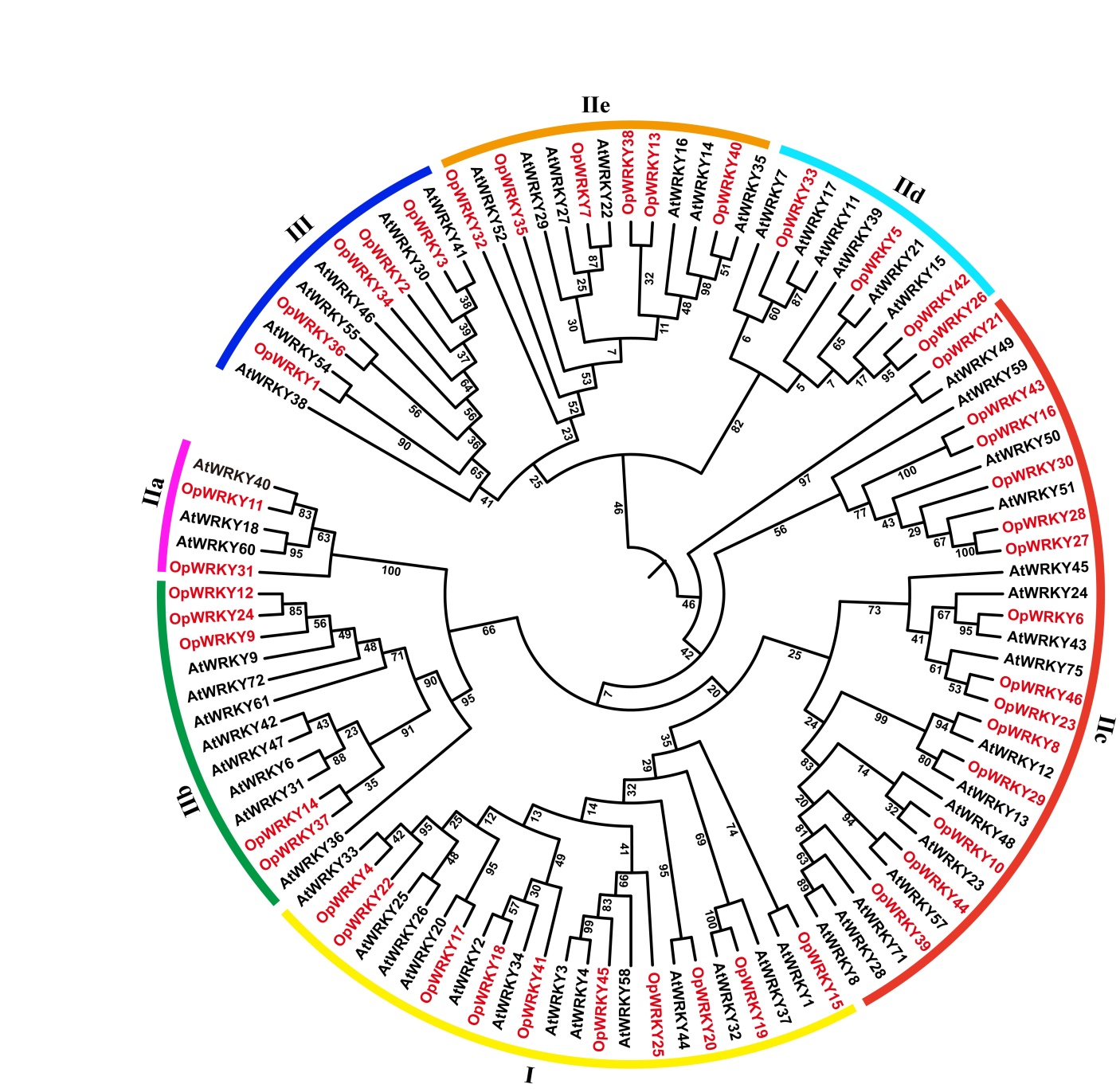


Supplementary **Figure 2.** Phylogenetic trees depicting WRKY family members in *A. thaliana* and *O. pumila*. The WRKY proteins were classified into 7 subfamilies and distinguished by different colors.


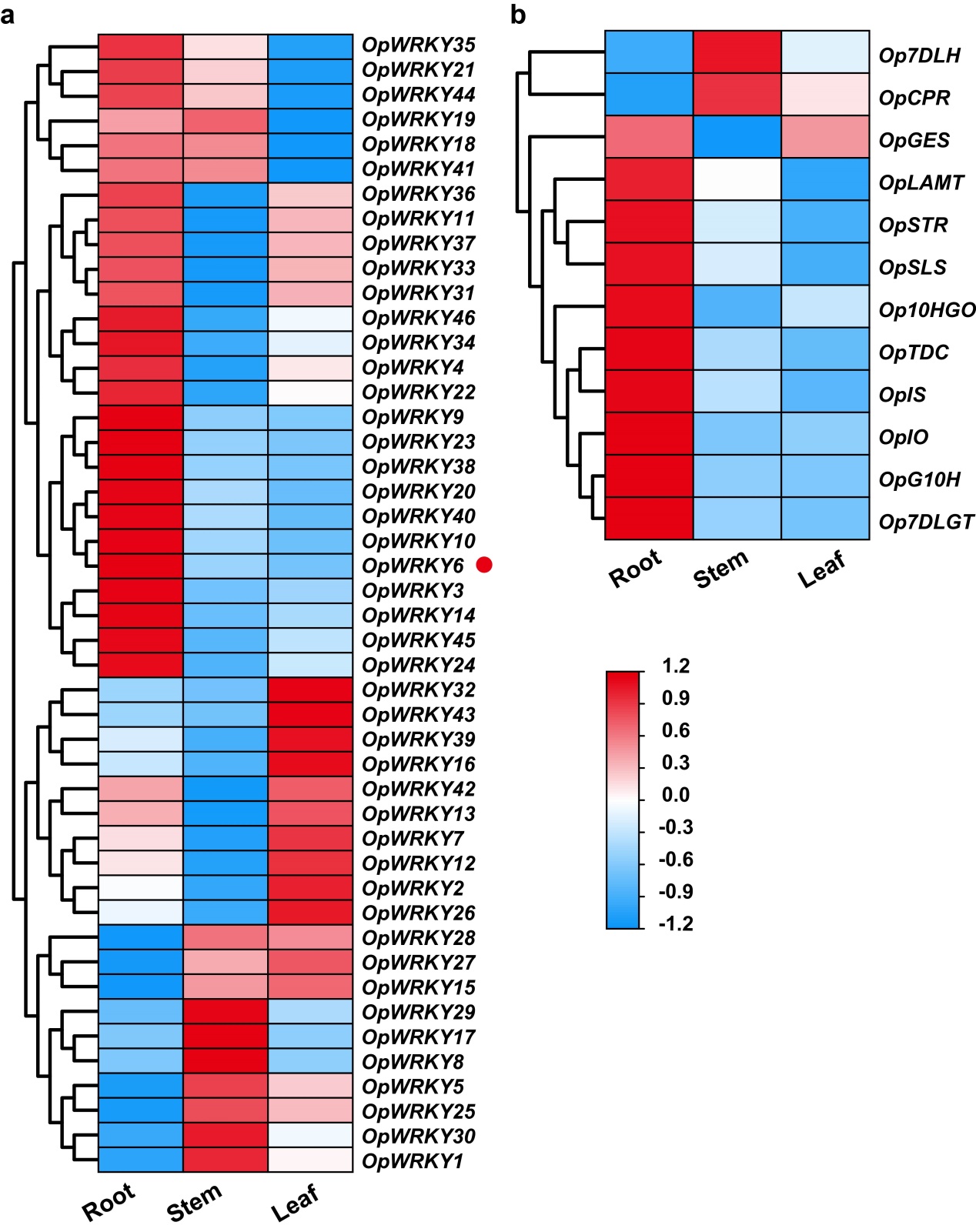


Supplementary **Figure 3.** Expression patterns of *OpWRKY* and camptothecin biostnthetic pathway genes. a. Heatmap showing *OpWRKY* gene expression in different organs. b. Expression profiles of camptothecin biostnthetic pathway genes in different organs.


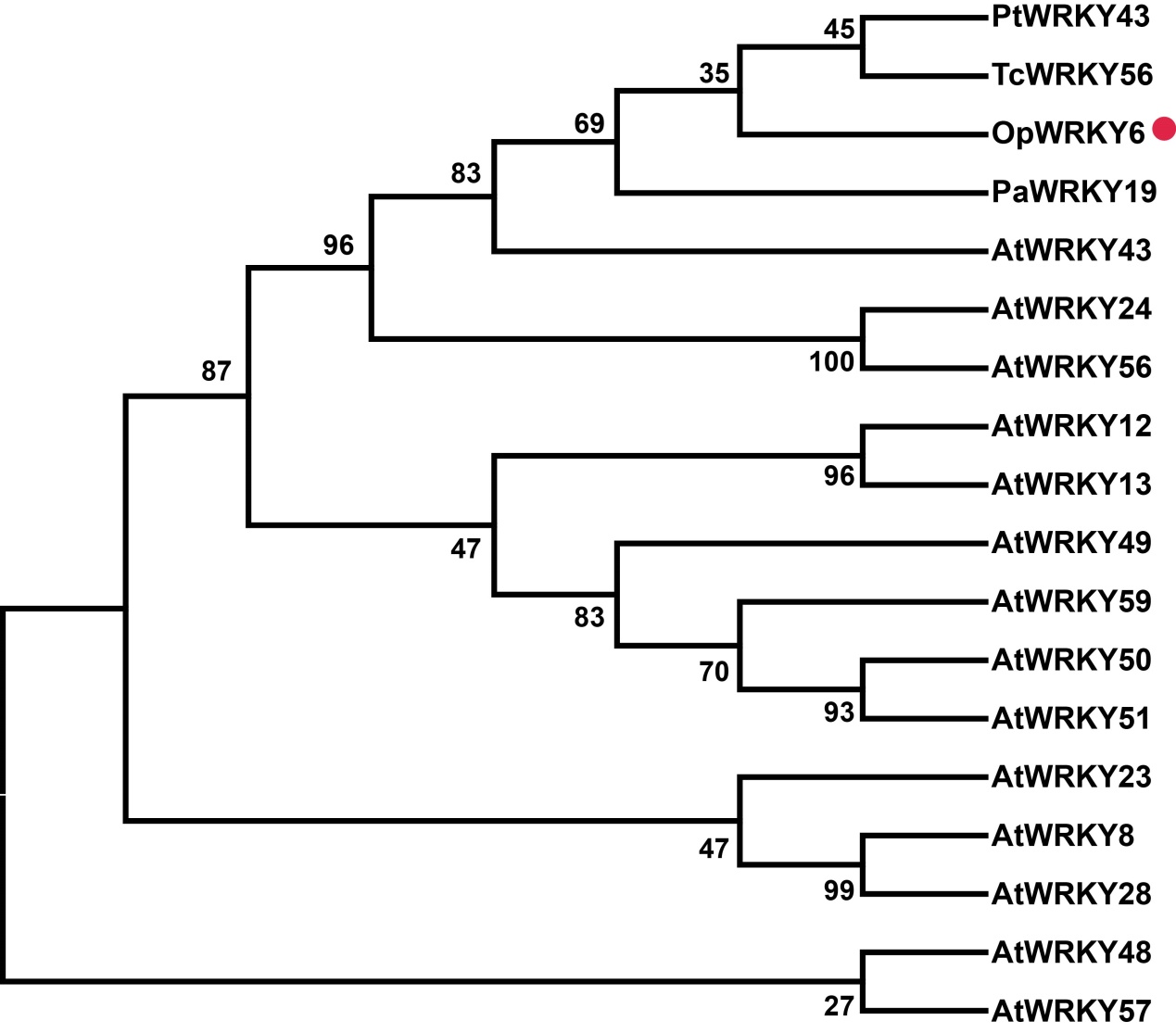


Supplementary **Figure 4.** Phylogenetic analysis of OpWRKY6 protein from *O. pumila* and other plants.


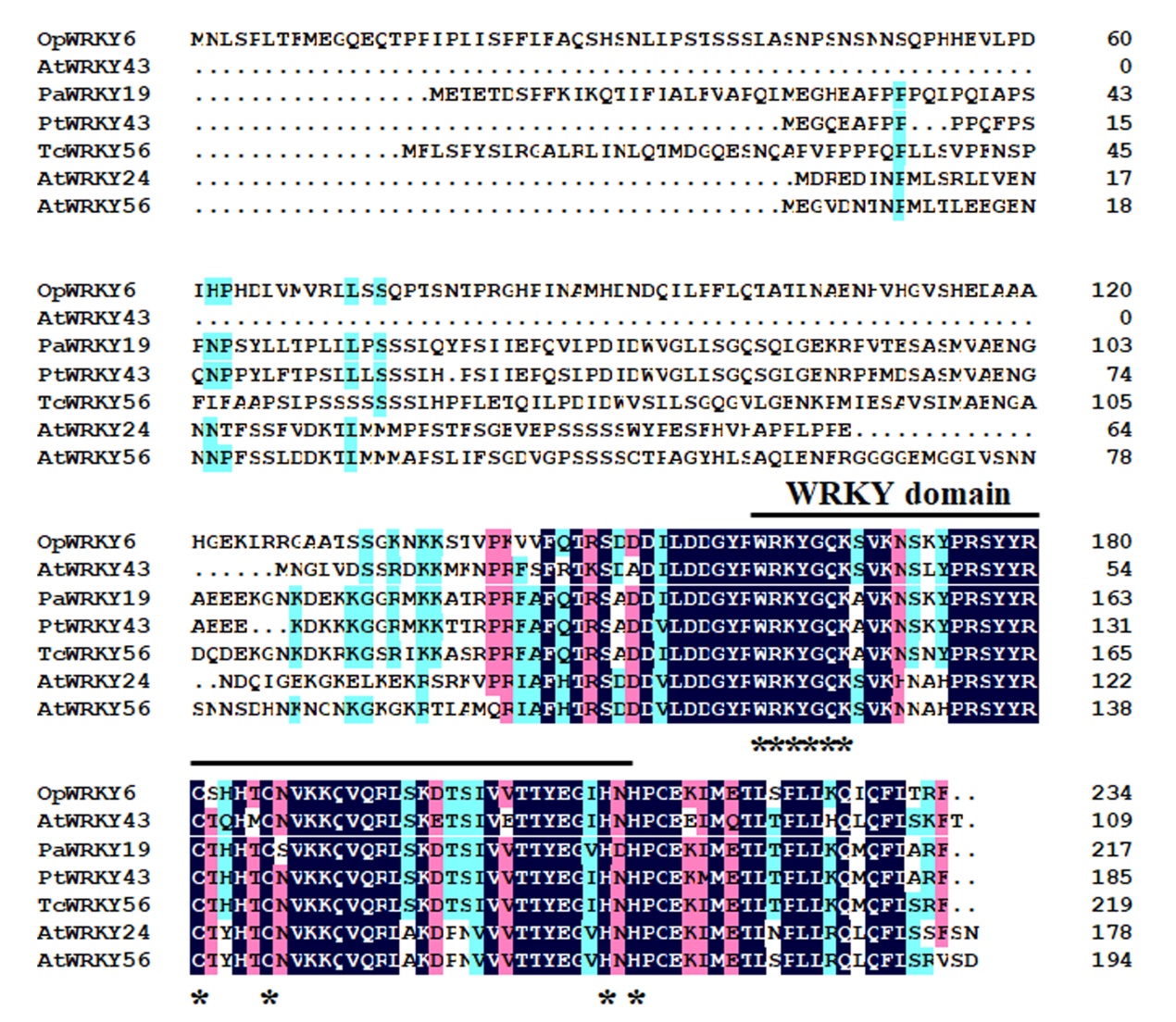


Supplementary **Figure 5.** Alignment of the protein sequences of OpWRKY6 and 6 related proteins belonging to IIc subfamily.


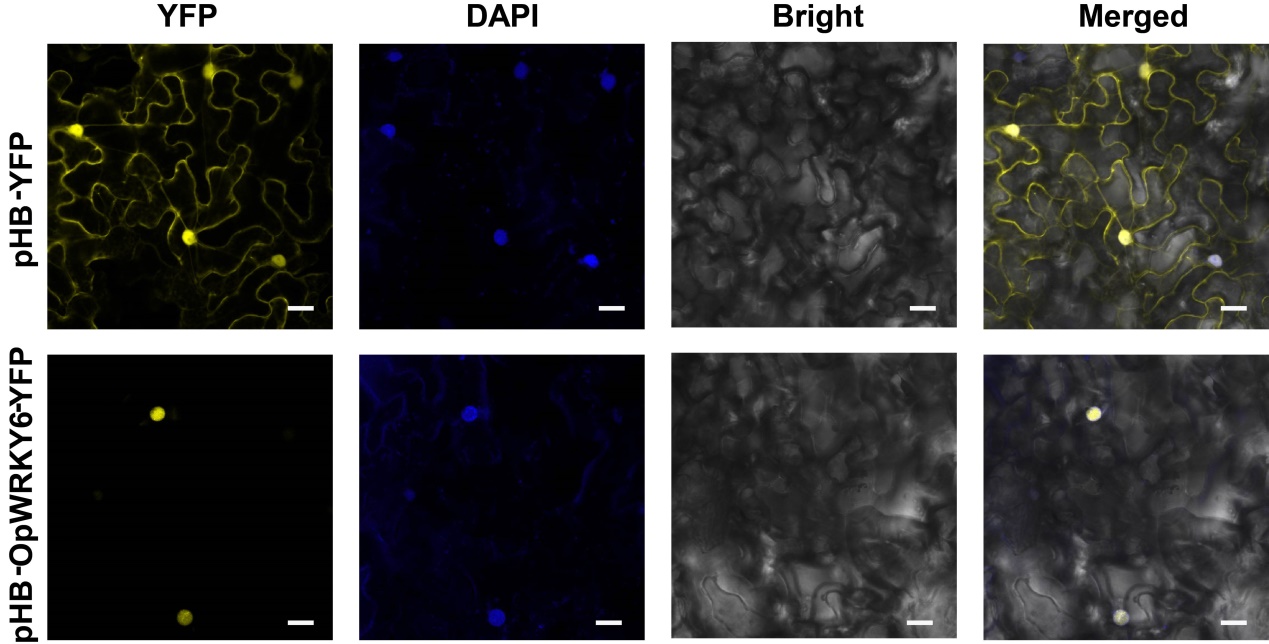


Supplementary **Figure 6.** Subcellular localization of OpWRKY6 in tobacco leaf epidermal cells. Colocalization of OpWRKY6-YFP and nuclei was determined by DAPI staining. YFP was used as a negative control. Scale bars, 20 µm.


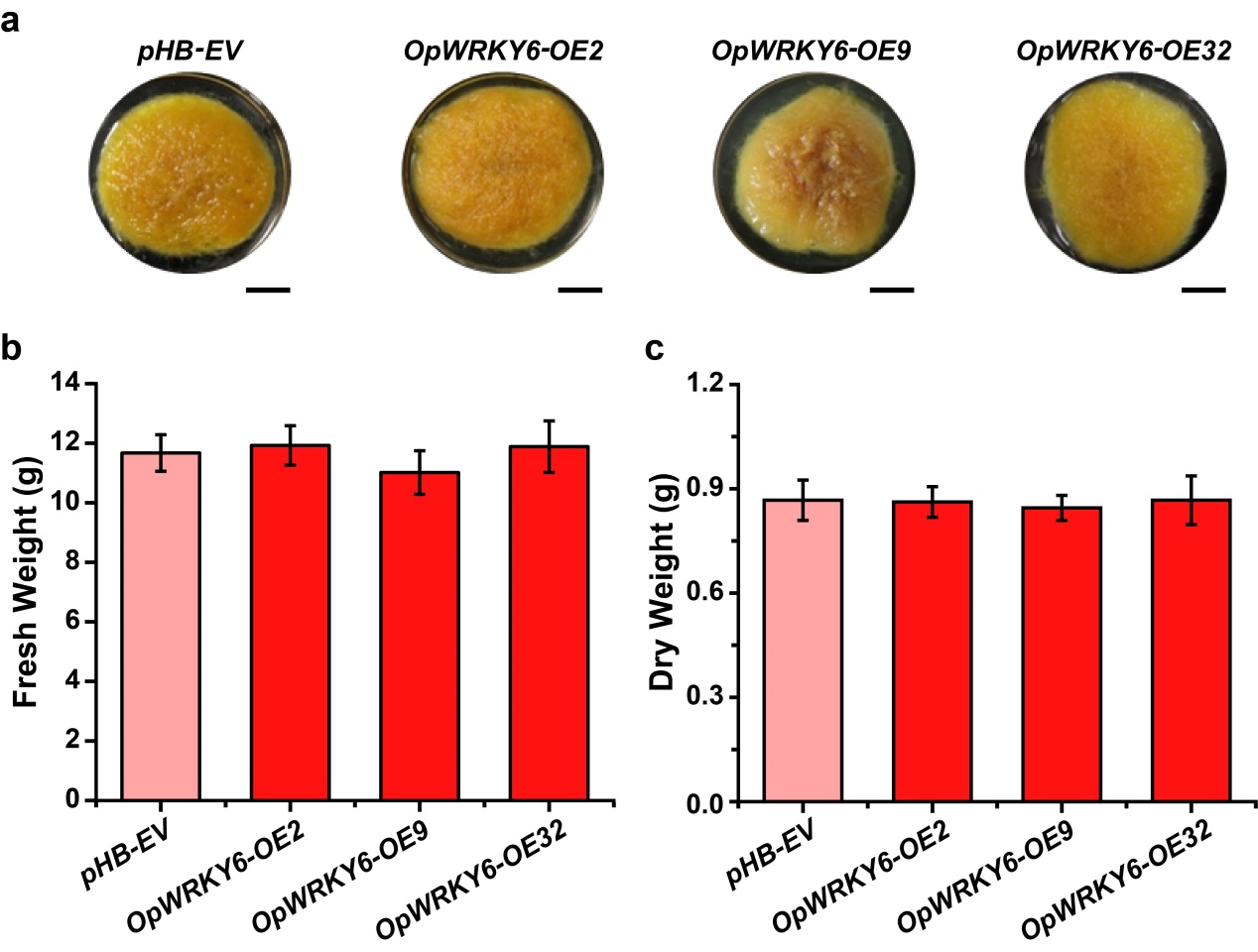


Supplementary **Figure 7.** a. The phenotype of the *OpWRKY6-OE* transgenic hairy root lines (scale bars, 1 cm). b-c. The fresh and dry weight of *OpWRKY6-OE* transgenic hairy root lines, respectively.


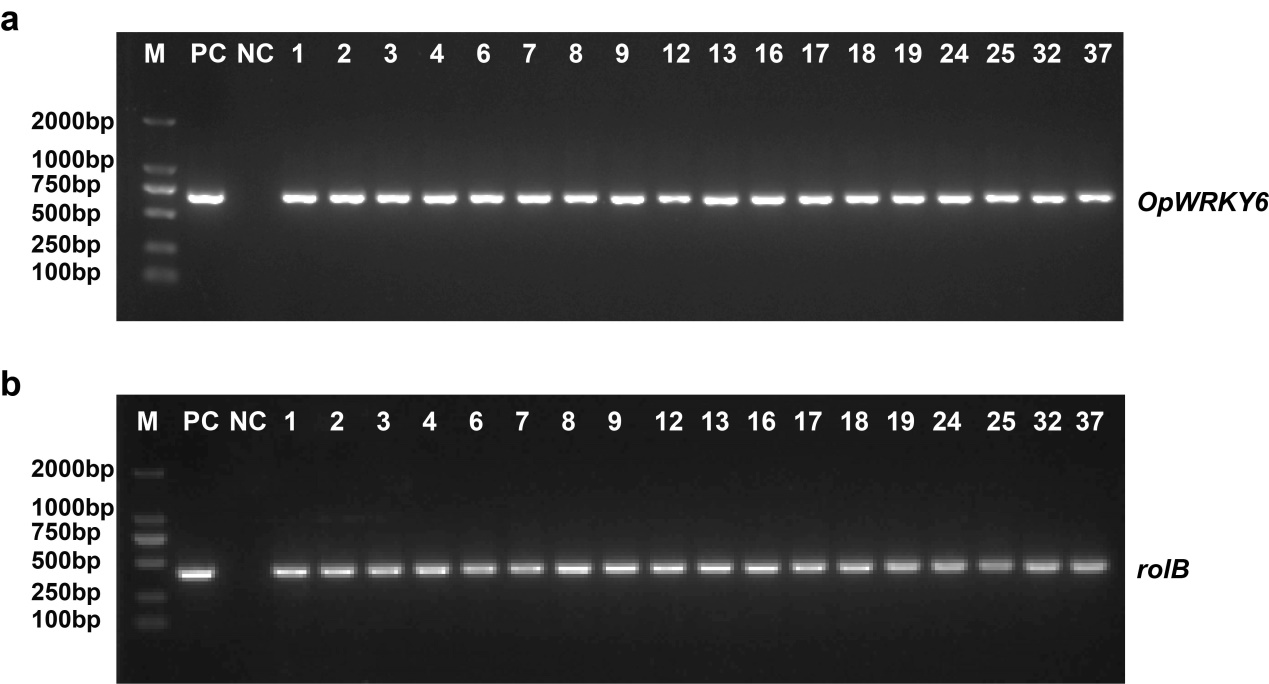


Supplementary **Figure 8.** Identification of positive OpWRKY6-KO transgenic hairy root lines by PCR. a. The *OpWRKY6* gene was detected by the primers OpWRKY6-F1 and OpWRKY6-R_531_. b. The rolB gene was used to confirm that the hairy roots were infected with Agrobacterium.


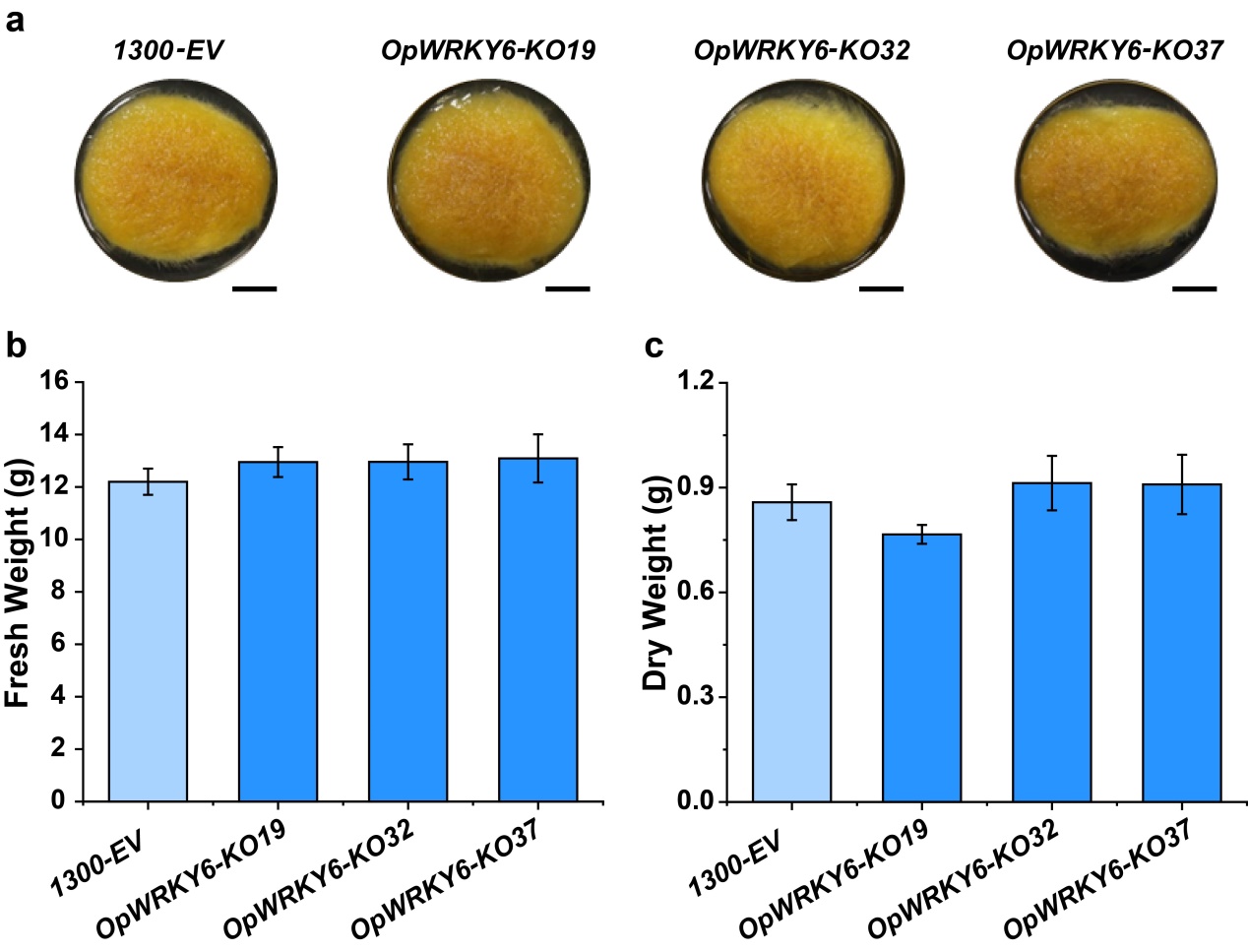


Supplementary **Figure 9.** a. The phenotype of the *OpWRKY6-KO* transgenic hairy root lines (scale bars: 1 cm). b-c. The fresh and dry weight of *OpWRKY6-KO* transgenic hairy root lines, respectively.


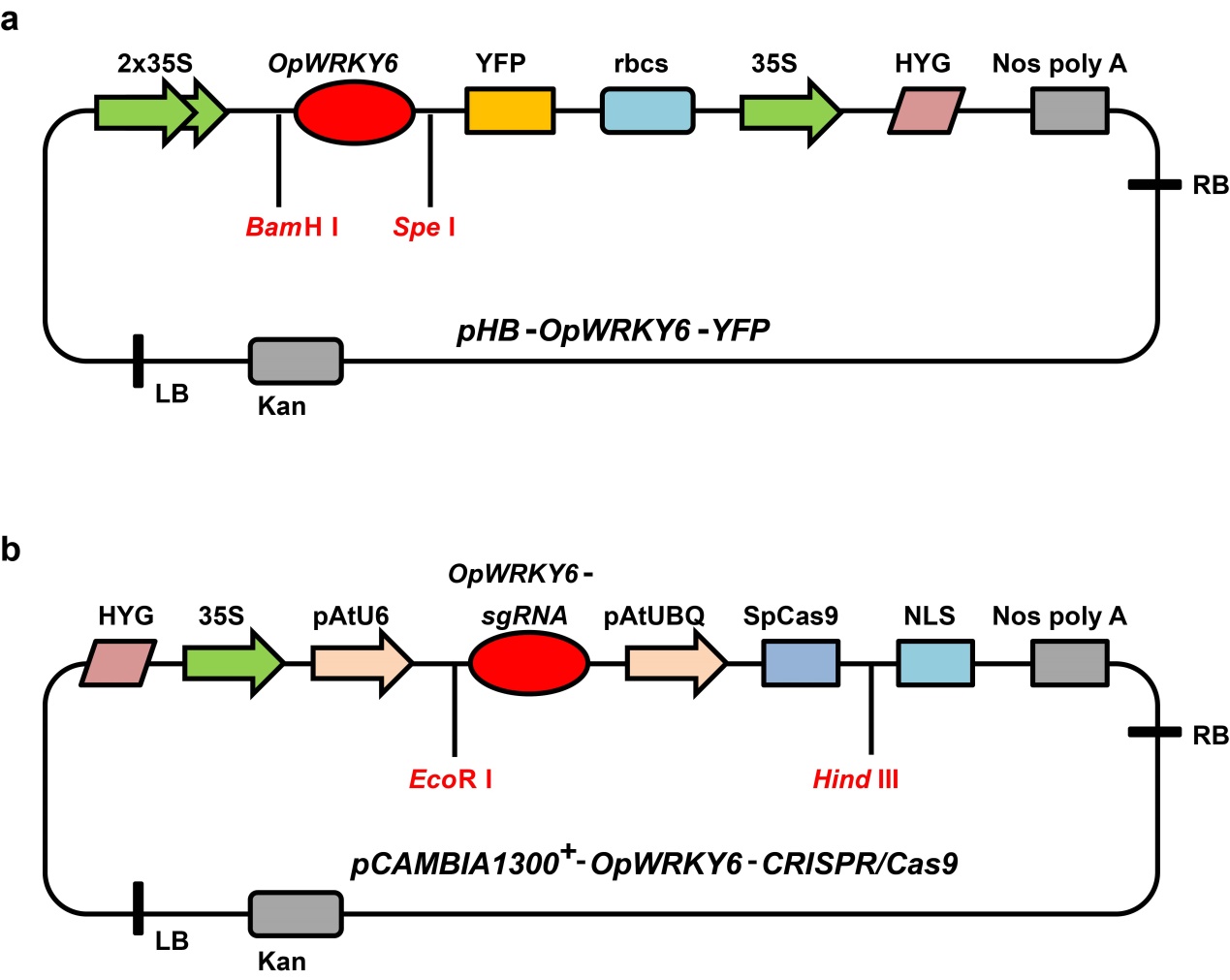


Supplementary **Figure 10.** The vector construction map. a. Construction of the *pHB-OpWRKY6* vector (*OpWRKY6-OE*). b. Construction of the *pCAMBIA1300^+^-OpWRKY6-CRISPR/Cas9* vector (*OpWRKY6-KO*).

**Table S1.** List of the identified *OpWRKY* genes and their related information.

| **Gene ID** | **Gene name** | **Chr** | **Start** | **Stop** | **Strand** | **Exon number** | **CDS (bp)** | **Size (aa)** | **MWs (Da)** | **pI** | **Loc** | **Gene Name in the Literature** |
| --- | --- | --- | --- | --- | --- | --- | --- | --- | --- | --- | --- | --- |
| Opuchr09_g0007470-1.1 | *OpWRKY1* | Opu_chr09 | 4,611,915 | 4,614,012 | + | 3 | 987 | 328 | 36,859.36 | 5.88 | nucleus | *OpWRKY1** |
| Opuchr02_g0001210-1.1 | *OpWRKY2* | Opu_chr02 | 782,883 | 785,060 | + | 3 | 1071 | 356 | 39,808.78 | 5.48 | nucleus | *OpWRKY2** |
| Opuchr09_g0002440-1.1 | *OpWRKY3* | Opu_chr09 | 1,498,467 | 1,499,797 | + | 3 | 1128 | 375 | 41,878.04 | 5.67 | nucleus | *OpWRKY3** |
| Opuchr01_g0002050-1.1 | *OpWRKY4* | Opu_chr01 | 1,032,577 | 1,035,149 | + | 5 | 1680 | 559 | 61,472.73 | 8.71 | nucleus | *OpWRKY28* |
| Opuchr01_g0071450-1.1 | *OpWRKY5* | Opu_chr01 | 34,503,123 | 34,501,512 | - | 3 | 1053 | 350 | 39,316.53 | 9.59 | nucleus | *OpWRKY20* |
| Opuchr02_g0001960-1.1 | *OpWRKY6* | Opu_chr02 | 1,211,296 | 1,209,850 | - | 2 | 705 | 234 | 26,291.60 | 9.12 | nucleus | *OpWRKY14* |
| Opuchr02_g0002010-1.1 | *OpWRKY7* | Opu_chr02 | 1,229,449 | 1,227,961 | - | 3 | 1065 | 354 | 38,564.59 | 5.92 | nucleus | *OpWRKY23* |
| Opuchr02_g0007230-1.1 | *OpWRKY8* | Opu_chr02 | 4,507,145 | 4,511,491 | + | 4 | 732 | 243 | 27,660.24 | 7.26 | nucleus | *-* |
| Opuchr02_g0069690-1.1 | *OpWRKY9* | Opu_chr02 | 37,438,979 | 37,441,068 | + | 5 | 1584 | 527 | 57,280.55 | 5.80 | nucleus | *OpWRKY6* |
| Opuchr03_g0000900-1.1 | *OpWRKY10* | Opu_chr03 | 559,342 | 557,652 | - | 3 | 1140 | 379 | 41,537.44 | 5.87 | nucleus | *OpWRKY17* |
| Opuchr04_g0006520-1.1 | *OpWRKY11* | Opu_chr04 | 3,532,198 | 3,533,801 | + | 5 | 945 | 314 | 34,863.19 | 8.55 | nucleus | *OpWRKY4* |
| Opuchr04_g0061040-1.1 | *OpWRKY12* | Opu_chr04 | 29,944,546 | 29,948,645 | + | 5 | 1815 | 604 | 65,777.14 | 7.16 | nucleus | *-* |
| Opuchr05_g0001510-1.1 | *OpWRKY13* | Opu_chr05 | 781,903 | 779,678 | - | 3 | 855 | 284 | 30,572.53 | 5.26 | nucleus | *OpWRKY24* |
| Opuchr05_g0010260-1.1 | *OpWRKY14* | Opu_chr05 | 5,402,983 | 5,400,233 | - | 5 | 1938 | 645 | 70,540.98 | 6.15 | nucleus | *OpWRKY8* |
| Opuchr05_g0039720-1.1 | *OpWRKY15* | Opu_chr05 | 17,645,223 | 17,648,283 | + | 4 | 1665 | 554 | 60,874.98 | 6.49 | nucleus | *OpWRKY25* |
| Opuchr05_g0052440-1.1 | *OpWRKY16* | Opu_chr05 | 23,437,601 | 23,434,804 | - | 3 | 684 | 227 | 26,044.99 | 6.18 | nucleus | *-* |
| Opuchr05_g0065400-1.1 | *OpWRKY17* | Opu_chr05 | 30,397,588 | 30,401,780 | + | 6 | 1851 | 616 | 66,936.53 | 6.40 | nucleus | *OpWRKY32* |
| Opuchr05_g0067290-1.1 | *OpWRKY18* | Opu_chr05 | 31,332,315 | 31,328,723 | - | 5 | 2,211 | 736 | 79,041.18 | 6.01 | nucleus | *OpWRKY27* |
| Opuchr06_g0105040-1.1 | *OpWRKY19* | Opu_chr06 | 50,289,572 | 50,294,319 | + | 5 | 1587 | 528 | 57,480.49 | 5.60 | nucleus | *OpWRKY29* |
| Opuchr06_g0116050-1.1 | *OpWRKY20* | Opu_chr06 | 57,240,042 | 57,242,358 | + | 5 | 1365 | 454 | 50,332.72 | 8.73 | nucleus | *OpWRKY30* |
| Opuchr06_g0117310-1.1 | *OpWRKY21* | Opu_chr06 | 57,961,856 | 57,960,634 | - | 3 | 993 | 330 | 37,083.97 | 5.24 | nucleus | *-* |
| Opuchr06_g0119930-1.1 | *OpWRKY22* | Opu_chr06 | 59,459,974 | 59,457,369 | - | 5 | 1752 | 583 | 64,367.61 | 8.39 | nucleus | *OpWRKY33* |
| Opuchr07_g0000470-1.1 | *OpWRKY23* | Opu_chr07 | 274,966 | 276,613 | + | 2 | 630 | 209 | 23,907.01 | 9.33 | nucleus | *-* |
| Opuchr07_g0003900-1.1 | *OpWRKY24* | Opu_chr07 | 2,134,979 | 2,131,731 | - | 4 | 1779 | 592 | 63,698.51 | 5.48 | nucleus | *OpWRKY9* |
| Opuchr07_g0004690-1.1 | *OpWRKY25* | Opu_chr07 | 2,625,874 | 2,622,760 | - | 4 | 1584 | 527 | 57,247.07 | 8.07 | nucleus | *OpWRKY26* |
| Opuchr07_g0008380-1.1 | *OpWRKY26* | Opu_chr07 | 4,656,914 | 4,655,633 | - | 3 | 1059 | 352 | 38,657.88 | 9.13 | nucleus | *OpWRKY18* |
| Opuchr07_g0076590-1.1 | *OpWRKY27* | Opu_chr07 | 35,342,198 | 35,336,367 | - | 3 | 654 | 217 | 24,567.82 | 8.82 | nucleus | *-* |
| Opuchr07_g0076660-1.1 | *OpWRKY28* | Opu_chr07 | 35,380,446 | 35,374,537 | - | 3 | 654 | 217 | 24,604.83 | 8.54 | nucleus | *-* |
| Opuchr07_g0077110-1.1 | *OpWRKY29* | Opu_chr07 | 35,627,733 | 35,631,063 | + | 3 | 669 | 222 | 25,509.62 | 8.85 | nucleus | *OpWRKY11* |
| Opuchr08_g0002460-1.1 | *OpWRKY30* | Opu_chr08 | 1,420,103 | 1,422,592 | + | 3 | 831 | 276 | 31,470.38 | 7.14 | nucleus | *OpWRKY15* |
| Opuchr08_g0009150-1.1 | *OpWRKY31* | Opu_chr08 | 5,059,683 | 5,057,272 | - | 5 | 921 | 306 | 34,446.42 | 6.10 | nucleus | *OpWRKY5* |
| Opuchr08_g0016990-1.1 | *OpWRKY32* | Opu_chr08 | 9,644,016 | 9,644,957 | + | 1 | 942 | 313 | 36,270.24 | 4.77 | nucleus | *-* |
| Opuchr08_g0025550-1.1 | *OpWRKY33* | Opu_chr08 | 13,113,636 | 13,111,976 | - | 3 | 1026 | 341 | 36,508.51 | 9.66 | nucleus | *OpWRKY19* |
| Opuchr08_g0083550-1.1 | *OpWRKY34* | Opu_chr08 | 37,804,145 | 37,802,692 | - | 3 | 1077 | 358 | 40,162.41 | 5.63 | nucleus | *OpWRKY31* |
| Opuchr09_g0003060-1.1 | *OpWRKY35* | Opu_chr09 | 1,876,787 | 1,875,469 | - | 3 | 1086 | 361 | 40,559.09 | 5.38 | nucleus | *-* |
| Opuchr09_g0007480-1.1 | *OpWRKY36* | Opu_chr09 | 4,621,497 | 4,618,954 | - | 3 | 1203 | 400 | 44,242.42 | 6.35 | nucleus | *-* |
| Opuchr09_g0010890-1.1 | *OpWRKY37* | Opu_chr09 | 6,715,129 | 6,717,330 | + | 6 | 1674 | 557 | 60,369.38 | 6.55 | nucleus | *OpWRKY7* |
| Opuchr09_g0018710-1.1 | *OpWRKY38* | Opu_chr09 | 11,561,088 | 11,562,283 | + | 3 | 957 | 318 | 35,732.19 | 5.17 | nucleus | *-* |
| Opuchr09_g0024990-1.1 | *OpWRKY39* | Opu_chr09 | 14,830,743 | 14,832,335 | + | 3 | 978 | 325 | 36,557.78 | 6.57 | nucleus | *OpWRKY13* |
| Opuchr09_g0089700-1.1 | *OpWRKY40* | Opu_chr09 | 40,046,973 | 40,049,605 | + | 3 | 1407 | 468 | 50,797.09 | 5.28 | nucleus | *OpWRKY22* |
| Opuchr09_g0099250-1.1 | *OpWRKY41* | Opu_chr09 | 45,930,002 | 45,933,337 | + | 5 | 2358 | 785 | 86,032.35 | 5.36 | nucleus | *-* |
| Opuchr10_g0007130-1.1 | *OpWRKY42* | Opu_chr10 | 3,764,703 | 3,763,367 | - | 3 | 1092 | 363 | 39,757.94 | 9.53 | nucleus | *OpWRKY34* |
| Opuchr10_g0010270-1.1 | *OpWRKY43* | Opu_chr10 | 5,717,170 | 5,714,326 | - | 3 | 684 | 227 | 25,857.64 | 6.03 | nucleus | *-* |
| Opuchr11_g0080910-1.1 | *OpWRKY44* | Opu_chr11 | 35,694,705 | 35,691,794 | - | 3 | 1035 | 344 | 37,556.48 | 5.45 | nucleus | *OpWRKY10* |
| Opuchr11_g0081300-1.1 | *OpWRKY45* | Opu_chr11 | 35,952,347 | 35,956,356 | + | 4 | 1557 | 518 | 56,623.44 | 6.04 | nucleus | *-* |
| Opuchr11_g0088100-1.1 | *OpWRKY46* | Opu_chr11 | 39,906,019 | 39,903,125 | - | 2 | 588 | 195 | 21,841.37 | 9.24 | nucleus | *OpWRKY16* |

Note: AA, amino acid residues; Chr, chromosome; MW, molecular weight; pI, Isoelectric point; Loc, subcellular location.

**Table S2.** The Blastp results of all OpWRKYs with previously published transcriptome data.

| **Query id**  **(In this study)** | **Subject id** | **% identity** | **Alignment length** | **Mismatches** | **Gap opens** | **Q. start** | **Q. end** | **S. start** | **S. end** | **E-value** | **Bit score** |
| --- | --- | --- | --- | --- | --- | --- | --- | --- | --- | --- | --- |
| OpWRKY1 | OpWRKY1 | 99.39 | 328 | 2 | 0 | 1 | 328 | 1 | 328 | 0 | 681 |
| OpWRKY2 | OpWRKY2 | 100 | 356 | 0 | 0 | 1 | 356 | 1 | 356 | 0 | 744 |
| OpWRKY3 | OpWRKY3 | 99.47 | 375 | 2 | 0 | 1 | 375 | 1 | 375 | 0 | 777 |
| OpWRKY4 | OpWRKY28 | 99.82 | 559 | 1 | 0 | 1 | 559 | 1 | 559 | 0 | 1151 |
| OpWRKY5 | OpWRKY20 | 98.57 | 350 | 5 | 0 | 1 | 350 | 1 | 350 | 0 | 716 |
| OpWRKY6 | OpWRKY14 | 100 | 234 | 0 | 0 | 1 | 234 | 1 | 234 | 1.00E-178 | 488 |
| OpWRKY7 | OpWRKY23 | 100 | 354 | 0 | 0 | 1 | 354 | 1 | 354 | 0 | 729 |
| OpWRKY9 | OpWRKY6 | 100 | 518 | 0 | 0 | 10 | 527 | 1 | 518 | 0 | 1073 |
| OpWRKY10 | OpWRKY17 | 100 | 379 | 0 | 0 | 1 | 379 | 1 | 379 | 0 | 776 |
| OpWRKY11 | OpWRKY4 | 100 | 314 | 0 | 0 | 1 | 314 | 1 | 314 | 0 | 652 |
| OpWRKY13 | OpWRKY24 | 98.74 | 159 | 2 | 0 | 1 | 159 | 1 | 159 | 6.00E-117 | 329 |
| OpWRKY14 | OpWRKY8 | 100 | 645 | 0 | 0 | 1 | 645 | 1 | 645 | 0 | 1342 |
| OpWRKY15 | OpWRKY25 | 99.64 | 554 | 1 | 1 | 1 | 554 | 1 | 553 | 0 | 1134 |
| OpWRKY17 | OpWRKY32 | 99.03 | 619 | 3 | 1 | 1 | 616 | 1 | 619 | 0 | 1269 |
| OpWRKY18 | OpWRKY27 | 99.86 | 736 | 1 | 0 | 1 | 736 | 1 | 736 | 0 | 1515 |
| OpWRKY19 | OpWRKY29 | 100 | 528 | 0 | 0 | 1 | 528 | 1 | 528 | 0 | 1098 |
| OpWRKY20 | OpWRKY30 | 98.46 | 454 | 0 | 1 | 1 | 454 | 1 | 447 | 0 | 926 |
| OpWRKY22 | OpWRKY33 | 99.83 | 583 | 1 | 0 | 1 | 583 | 1 | 583 | 0 | 1202 |
| OpWRKY24 | OpWRKY9 | 99.46 | 557 | 1 | 1 | 36 | 592 | 1 | 555 | 0 | 1139 |
| OpWRKY25 | OpWRKY26 | 100 | 527 | 0 | 0 | 1 | 527 | 1 | 527 | 0 | 1094 |
| OpWRKY26 | OpWRKY18 | 99.15 | 352 | 3 | 0 | 1 | 352 | 1 | 352 | 0 | 713 |
| OpWRKY29 | OpWRKY11 | 96.85 | 222 | 0 | 1 | 8 | 222 | 1 | 222 | 2.00E-164 | 449 |
| OpWRKY30 | OpWRKY15 | 100 | 219 | 0 | 0 | 58 | 276 | 1 | 219 | 4.00E-167 | 458 |
| OpWRKY31 | OpWRKY5 | 99.67 | 306 | 0 | 1 | 1 | 306 | 1 | 305 | 0 | 633 |
| OpWRKY33 | OpWRKY19 | 100 | 341 | 0 | 0 | 1 | 341 | 1 | 341 | 0 | 700 |
| OpWRKY34 | OpWRKY31 | 100 | 358 | 0 | 0 | 1 | 358 | 1 | 358 | 0 | 749 |
| OpWRKY37 | OpWRKY7 | 100 | 557 | 0 | 0 | 1 | 557 | 1 | 557 | 0 | 1163 |
| OpWRKY39 | OpWRKY13 | 100 | 325 | 0 | 0 | 1 | 325 | 1 | 325 | 0 | 676 |
| OpWRKY40 | OpWRKY22 | 100 | 440 | 0 | 0 | 29 | 468 | 1 | 440 | 0 | 917 |
| OpWRKY42 | OpWRKY34 | 99.72 | 363 | 1 | 0 | 1 | 363 | 1 | 363 | 0 | 751 |
| OpWRKY44 | OpWRKY10 | 100 | 344 | 0 | 0 | 1 | 344 | 1 | 344 | 0 | 714 |
| OpWRKY46 | OpWRKY16 | 100 | 195 | 0 | 0 | 1 | 195 | 1 | 195 | 4.00E-149 | 408 |

**Table S3.** Synteny analysis of *WRKY* genes between *O. pumila* and *Arabidopsis*, *O. sativa*, *V. vinifera* and *C. eugenioides*, respectively.

| **Gene_ID** | **Op_Chrom** | **Gene_name** | **Gene_ID** | **Chrom** | **Gene_name** | **Species** | **Type** |
| --- | --- | --- | --- | --- | --- | --- | --- |
| Opuchr01_g0002050-1.1 | Opu_chr01 | *OpWRKY1* | AT2G30250.1.TAIR10 | Chr2 | *AtWRKY25* | *Arabidopsis thaliana* | I |
| Opuchr01_g0071450-1.1 | Opu_chr01 | *OpWRKY2* | AT2G30590.1.TAIR10 | Chr2 | *AtWRKY21* | *Arabidopsis thaliana* | IId |
| Opuchr01_g0002050-1.1 | Opu_chr01 | *OpWRKY1* | AT5G07100.1.TAIR10 | Chr5 | *AtWRKY26* | *Arabidopsis thaliana* | I |
| Opuchr02_g0001960-1.1 | Opu_chr02 | *OpWRKY4* | AT1G64000.1.TAIR10 | Chr1 | *AtWRKY56* | *Arabidopsis thaliana* | IIc |
| Opuchr02_g0007230-1.1 | Opu_chr02 | *OpWRKY6* | AT2G44745.1.TAIR10 | Chr2 | *AtWRKY12* | *Arabidopsis thaliana* | IIc |
| Opuchr02_g0001960-1.1 | Opu_chr02 | *OpWRKY4* | AT2G46130.1.TAIR10 | Chr2 | *AtWRKY43* | *Arabidopsis thaliana* | IIc |
| Opuchr02_g0001210-1.1 | Opu_chr02 | *OpWRKY3* | AT2G46400.1.TAIR10 | Chr2 | *AtWRKY46* | *Arabidopsis thaliana* | III |
| Opuchr02_g0002010-1.1 | Opu_chr02 | *OpWRKY5* | AT4G01250.1.TAIR10 | Chr4 | *AtWRKY22* | *Arabidopsis thaliana* | IIe |
| Opuchr02_g0002010-1.1 | Opu_chr02 | *OpWRKY5* | AT4G23550.1.TAIR10 | Chr4 | *AtWRKY29* | *Arabidopsis thaliana* | IIe |
| Opuchr02_g0001960-1.1 | Opu_chr02 | *OpWRKY4* | AT5G41570.1.TAIR10 | Chr5 | *AtWRKY24* | *Arabidopsis thaliana* | IIc |
| Opuchr03_g0000900-1.1 | Opu_chr03 | *OpWRKY8* | AT2G47260.1.TAIR10 | Chr2 | *AtWRKY23* | *Arabidopsis thaliana* | IIc |
| Opuchr03_g0000900-1.1 | Opu_chr03 | *OpWRKY8* | AT5G49520.1.TAIR10 | Chr5 | *AtWRKY48* | *Arabidopsis thaliana* | IIc |
| Opuchr04_g0006520-1.1 | Opu_chr04 | *OpWRKY9* | AT1G80840.1.TAIR10 | Chr1 | *AtWRKY40* | *Arabidopsis thaliana* | IIa |
| Opuchr04_g0061040-1.1 | Opu_chr04 | *OpWRKY10* | AT1G69810.1.TAIR10 | Chr1 | *AtWRKY36* | *Arabidopsis thaliana* | IIb |
| Opuchr04_g0006520-1.1 | Opu_chr04 | *OpWRKY9* | AT2G25000.1.TAIR10 | Chr2 | *AtWRKY60* | *Arabidopsis thaliana* | IIa |
| Opuchr04_g0006520-1.1 | Opu_chr04 | *OpWRKY9* | AT4G31800.1.TAIR10 | Chr4 | *AtWRKY18* | *Arabidopsis thaliana* | IIa |
| Opuchr05_g0001510-1.1 | Opu_chr05 | *OpWRKY11* | AT3G58710.1.TAIR10 | Chr3 | *AtWRKY69* | *Arabidopsis thaliana* | IIe |
| Opuchr05_g0065400-1.1 | Opu_chr05 | *OpWRKY15* | AT4G26640.2.TAIR10 | Chr4 | *AtWRKY20* | *Arabidopsis thaliana* | I |
| Opuchr05_g0067290-1.1 | Opu_chr05 | *OpWRKY16* | AT4G26440.1.TAIR10 | Chr4 | *AtWRKY34* | *Arabidopsis thaliana* | I |
| Opuchr05_g0067290-1.1 | Opu_chr05 | *OpWRKY16* | AT5G56270.1.TAIR10 | Chr5 | *AtWRKY2* | *Arabidopsis thaliana* | I |
| Opuchr06_g0116050-1.1 | Opu_chr06 | *OpWRKY18* | AT2G37260.1.TAIR10 | Chr2 | *AtWRKY44* | *Arabidopsis thaliana* | I |
| Opuchr06_g0119930-1.1 | Opu_chr06 | *OpWRKY20* | AT2G38470.1.TAIR10 | Chr2 | *AtWRKY33* | *Arabidopsis thaliana* | I |
| Opuchr06_g0105040-1.1 | Opu_chr06 | *OpWRKY17* | AT4G30935.1.TAIR10 | Chr4 | *AtWRKY32* | *Arabidopsis thaliana* | I |
| Opuchr07_g0004690-1.1 | Opu_chr07 | *OpWRKY23* | AT1G13960.1.TAIR10 | Chr1 | *AtWRKY4* | *Arabidopsis thaliana* | I |
| Opuchr07_g0008380-1.1 | Opu_chr07 | *OpWRKY24* | AT2G23320.1.TAIR10 | Chr2 | *AtWRKY15* | *Arabidopsis thaliana* | IId |
| Opuchr07_g0076590-1.1 | Opu_chr07 | *OpWRKY25* | AT2G21900.1.TAIR10 | Chr2 | *AtWRKY59* | *Arabidopsis thaliana* | IIc |
| Opuchr07_g0004690-1.1 | Opu_chr07 | *OpWRKY23* | AT3G01080.1.TAIR10 | Chr3 | *AtWRKY58* | *Arabidopsis thaliana* | I |
| Opuchr07_g0000470-1.1 | Opu_chr07 | *OpWRKY21* | AT3G01970.1.TAIR10 | Chr3 | *AtWRKY45* | *Arabidopsis thaliana* | IIc |
| Opuchr07_g0077110-1.1 | Opu_chr07 | *OpWRKY27* | AT4G39410.1.TAIR10 | Chr4 | *AtWRKY13* | *Arabidopsis thaliana* | IIc |
| Opuchr07_g0003900-1.1 | Opu_chr07 | *OpWRKY22* | AT5G15130.1.TAIR10 | Chr5 | *AtWRKY72* | *Arabidopsis thaliana* | IIb |
| Opuchr07_g0076590-1.1 | Opu_chr07 | *OpWRKY25* | AT5G64810.1.TAIR10 | Chr5 | *AtWRKY51* | *Arabidopsis thaliana* | IIc |
| Opuchr08_g0083550-1.1 | Opu_chr08 | *OpWRKY32* | AT2G46400.1.TAIR10 | Chr2 | *AtWRKY46* | *Arabidopsis thaliana* | III |
| Opuchr08_g0083550-1.1 | Opu_chr08 | *OpWRKY32* | AT4G23810.1.TAIR10 | Chr4 | *AtWRKY53* | *Arabidopsis thaliana* | III |
| Opuchr08_g0083550-1.1 | Opu_chr08 | *OpWRKY32* | AT4G11070.1.TAIR10 | Chr4 | *AtWRKY41* | *Arabidopsis thaliana* | III |
| Opuchr08_g0002460-1.1 | Opu_chr08 | *OpWRKY28* | AT5G26170.1.TAIR10 | Chr5 | *AtWRKY50* | *Arabidopsis thaliana* | IIc |
| Opuchr09_g0018710-1.1 | Opu_chr09 | *OpWRKY38* | AT1G29280.1.TAIR10 | Chr1 | *AtWRKY65* | *Arabidopsis thaliana* | IIe |
| Opuchr09_g0010890-1.1 | Opu_chr09 | *OpWRKY37* | AT1G62300.1.TAIR10 | Chr1 | *AtWRKY6* | *Arabidopsis thaliana* | IIb |
| Opuchr09_g0089700-1.1 | Opu_chr09 | *OpWRKY40* | AT1G30650.1.TAIR10 | Chr1 | *AtWRKY14* | *Arabidopsis thaliana* | IIe |
| Opuchr09_g0007480-1.1 | Opu_chr09 | *OpWRKY36* | AT2G40740.1.TAIR10 | Chr2 | *AtWRKY55* | *Arabidopsis thaliana* | III |
| Opuchr09_g0007470-1.1 | Opu_chr09 | *OpWRKY35* | AT2G40750.1.TAIR10 | Chr2 | *AtWRKY54* | *Arabidopsis thaliana* | III |
| Opuchr09_g0002440-1.1 | Opu_chr09 | *OpWRKY33* | AT2G46400.1.TAIR10 | Chr2 | *AtWRKY46* | *Arabidopsis thaliana* | III |
| Opuchr09_g0007470-1.1 | Opu_chr09 | *OpWRKY35* | AT3G56400.1.TAIR10 | Chr3 | *AtWRKY70* | *Arabidopsis thaliana* | III |
| Opuchr09_g0010890-1.1 | Opu_chr09 | *OpWRKY37* | AT4G04450.1.TAIR10 | Chr4 | *AtWRKY42* | *Arabidopsis thaliana* | IIb |
| Opuchr09_g0024990-1.1 | Opu_chr09 | *OpWRKY39* | AT4G18170.1.TAIR10 | Chr4 | *AtWRKY28* | *Arabidopsis thaliana* | IIc |
| Opuchr09_g0010890-1.1 | Opu_chr09 | *OpWRKY37* | AT4G22070.1.TAIR10 | Chr4 | *AtWRKY31* | *Arabidopsis thaliana* | IIb |
| Opuchr09_g0003060-1.1 | Opu_chr09 | *OpWRKY34* | AT4G23550.1.TAIR10 | Chr4 | *AtWRKY29* | *Arabidopsis thaliana* | IIe |
| Opuchr09_g0002440-1.1 | Opu_chr09 | *OpWRKY33* | AT4G23810.1.TAIR10 | Chr4 | *AtWRKY53* | *Arabidopsis thaliana* | III |
| Opuchr09_g0003060-1.1 | Opu_chr09 | *OpWRKY34* | AT5G52830.1.TAIR10 | Chr5 | *AtWRKY27* | *Arabidopsis thaliana* | IIe |
| Opuchr09_g0002440-1.1 | Opu_chr09 | *OpWRKY33* | AT5G24110.1.TAIR10 | Chr5 | *AtWRKY30* | *Arabidopsis thaliana* | III |
| Opuchr11_g0080910-1.1 | Opu_chr11 | *OpWRKY44* | AT1G69310.1.TAIR10 | Chr1 | *AtWRKY57* | *Arabidopsis thaliana* | IIc |
| Opuchr11_g0081300-1.1 | Opu_chr11 | *OpWRKY45* | AT1G13960.1.TAIR10 | Chr1 | *AtWRKY4* | *Arabidopsis thaliana* | I |
| Opuchr11_g0081300-1.1 | Opu_chr11 | *OpWRKY45* | AT2G03340.1.TAIR10 | Chr2 | *AtWRKY3* | *Arabidopsis thaliana* | I |
| Opuchr11_g0088100-1.1 | Opu_chr11 | *OpWRKY46* | AT5G13080.1.TAIR10 | Chr5 | *AtWRKY75* | *Arabidopsis thaliana* | IIc |
| Opuchr01_g0002050-1.1 | Opu_chr01 | *OpWRKY1* | LOC_Os01g61080.1.MSUv7.0 | Chr1 | *-* | *Oryza sativa* | I |
| Opuchr01_g0071450-1.1 | Opu_chr01 | *OpWRKY2* | LOC_Os12g40570.1.MSUv7.0 | Chr12 | *-* | *Oryza sativa* | IId |
| Opuchr01_g0071450-1.1 | Opu_chr01 | *OpWRKY2* | LOC_Os03g53050.1.MSUv7.0 | Chr3 | *-* | *Oryza sativa* | IId |
| Opuchr01_g0071450-1.1 | Opu_chr01 | *OpWRKY2* | LOC_Os03g58420.1.MSUv7.0 | Chr3 | *-* | *Oryza sativa* | IId |
| Opuchr01_g0002050-1.1 | Opu_chr01 | *OpWRKY1* | LOC_Os05g39720.1.MSUv7.0 | Chr5 | *-* | *Oryza sativa* | I |
| Opuchr02_g0001210-1.1 | Opu_chr02 | *OpWRKY3* | LOC_Os01g46800.1.MSUv7.0 | Chr1 | *-* | *Oryza sativa* | III |
| Opuchr02_g0007230-1.1 | Opu_chr02 | *OpWRKY6* | LOC_Os02g43560.1.MSUv7.0 | Chr2 | *-* | *Oryza sativa* | IIc |
| Opuchr02_g0007230-1.1 | Opu_chr02 | *OpWRKY6* | LOC_Os04g46060.1.MSUv7.0 | Chr4 | *-* | *Oryza sativa* | IIc |
| Opuchr04_g0006520-1.1 | Opu_chr04 | *OpWRKY9* | LOC_Os02g08440.1.MSUv7.0 | Chr2 | *-* | *Oryza sativa* | IIa |
| Opuchr04_g0006520-1.1 | Opu_chr04 | *OpWRKY9* | LOC_Os06g44010.1.MSUv7.0 | Chr6 | *-* | *Oryza sativa* | IIa |
| Opuchr05_g0065400-1.1 | Opu_chr05 | *OpWRKY15* | LOC_Os07g39480.1.MSUv7.0 | Chr7 | *-* | *Oryza sativa* | I |
| Opuchr05_g0067290-1.1 | Opu_chr05 | *OpWRKY16* | LOC_Os08g38990.3.MSUv7.0 | Chr8 | *-* | *Oryza sativa* | I |
| Opuchr05_g0067290-1.1 | Opu_chr05 | *OpWRKY16* | LOC_Os09g30400.3.MSUv7.0 | Chr9 | *-* | *Oryza sativa* | I |
| Opuchr06_g0119930-1.1 | Opu_chr06 | *OpWRKY20* | LOC_Os01g61080.1.MSUv7.0 | Chr1 | *-* | *Oryza sativa* | I |
| Opuchr06_g0119930-1.1 | Opu_chr06 | *OpWRKY20* | LOC_Os05g39720.1.MSUv7.0 | Chr5 | *-* | *Oryza sativa* | I |
| Opuchr06_g0119930-1.1 | Opu_chr06 | *OpWRKY20* | LOC_Os05g27730.1.MSUv7.0 | Chr5 | *-* | *Oryza sativa* | I |
| Opuchr08_g0083550-1.1 | Opu_chr08 | *OpWRKY32* | LOC_Os01g46800.1.MSUv7.0 | Chr1 | *-* | *Oryza sativa* | III |
| Opuchr09_g0002440-1.1 | Opu_chr09 | *OpWRKY33* | LOC_Os01g46800.1.MSUv7.0 | Chr1 | *-* | *Oryza sativa* | III |
| Opuchr09_g0003060-1.1 | Opu_chr09 | *OpWRKY34* | LOC_Os01g43550.1.MSUv7.0 | Chr1 | *-* | *Oryza sativa* | IIe |
| Opuchr09_g0018710-1.1 | Opu_chr09 | *OpWRKY38* | LOC_Os01g54600.1.MSUv7.0 | Chr1 | *-* | *Oryza sativa* | IIe |
| Opuchr09_g0007470-1.1 | Opu_chr09 | *OpWRKY35* | LOC_Os03g21710.1.MSUv7.0 | Chr3 | *-* | *Oryza sativa* | III |
| Opuchr11_g0080910-1.1 | Opu_chr11 | *OpWRKY44* | LOC_Os03g55080.1.MSUv7.0 | Chr3 | *-* | *Oryza sativa* | IIc |
| Opuchr06_g0105040-1.1 | Opu_chr06 | *OpWRKY17* | VIT_204s0008g06600.1.v2.1 | Chr4 | *-* | *Vitis vinifera* | I |
| Opuchr06_g0119930-1.1 | Opu_chr06 | *OpWRKY20* | VIT_206s0004g07500.1.v2.1 | Chr6 | *-* | *Vitis vinifera* | I |
| Opuchr06_g0117310-1.1 | Opu_chr06 | *OpWRKY19* | VIT_208s0007g00570.1.v2.1 | Chr8 | *-* | *Vitis vinifera* | IIc |
| Opuchr06_g0119930-1.1 | Opu_chr06 | *OpWRKY20* | VIT_208s0058g00690.1.v2.1 | Chr8 | *-* | *Vitis vinifera* | I |
| Opuchr06_g0116050-1.1 | Opu_chr06 | *OpWRKY18* | VIT_208s0040g03070.3.v2.1 | Chr8 | *-* | *Vitis vinifera* | I |
| Opuchr07_g0003900-1.1 | Opu_chr07 | *OpWRKY22* | VIT_201s0026g01730.1.v2.1 | Chr1 | *-* | *Vitis vinifera* | IIb |
| Opuchr07_g0076590-1.1 | Opu_chr07 | *OpWRKY25* | VIT_207s0031g01710.1.v2.1 | Chr7 | *-* | *Vitis vinifera* | IIc |
| Opuchr07_g0077110-1.1 | Opu_chr07 | *OpWRKY27* | VIT_207s0031g01840.1.v2.1 | Chr7 | *-* | *Vitis vinifera* | IIc |
| Opuchr07_g0008380-1.1 | Opu_chr07 | *OpWRKY24* | VIT_207s0031g00080.1.v2.1 | Chr7 | *-* | *Vitis vinifera* | IId |
| Opuchr08_g0083550-1.1 | Opu_chr08 | *OpWRKY32* | VIT_202s0025g01280.1.v2.1 | Chr2 | *-* | *Vitis vinifera* | III |
| Opuchr08_g0002460-1.1 | Opu_chr08 | *OpWRKY28* | VIT_204s0008g01470.1.v2.1 | Chr4 | *-* | *Vitis vinifera* | IIc |
| Opuchr09_g0002440-1.1 | Opu_chr09 | *OpWRKY33* | VIT_202s0025g01280.1.v2.1 | Chr2 | *-* | *Vitis vinifera* | III |
| Opuchr09_g0003060-1.1 | Opu_chr09 | *OpWRKY34* | VIT_202s0025g00420.1.v2.1 | Chr2 | *-* | *Vitis vinifera* | IIe |
| Opuchr09_g0099250-1.1 | Opu_chr09 | *OpWRKY41* | VIT_204s0023g00470.2.v2.1 | Chr4 | *-* | *Vitis vinifera* | I |
| Opuchr09_g0007470-1.1 | Opu_chr09 | *OpWRKY35* | VIT_208s0058g01390.1.v2.1 | Chr8 | *-* | *Vitis vinifera* | III |
| Opuchr10_g0010270-1.1 | Opu_chr10 | *OpWRKY43* | VIT_207s0031g01710.1.v2.1 | Chr7 | *-* | *Vitis vinifera* | IIc |
| Opuchr10_g0007130-1.1 | Opu_chr10 | *OpWRKY42* | VIT_207s0031g00080.1.v2.1 | Chr7 | *-* | *Vitis vinifera* | IId |
| Opuchr11_g0088100-1.1 | Opu_chr11 | *OpWRKY46* | VIT_201s0010g03930.1.v2.1 | Chr1 | *-* | *Vitis vinifera* | IIc |
| Opuchr11_g0080910-1.1 | Opu_chr11 | *OpWRKY44* | VIT_201s0011g00720.1.v2.1 | Chr1 | *-* | *Vitis vinifera* | IIc |
| Opuchr01_g0002050-1.1 | Opu_chr01 | *OpWRKY1* | rna-XM_027319779.1 | Chr1 | *-* | *Coffea eugenioides* | I |
| Opuchr01_g0071450-1.1 | Opu_chr01 | *OpWRKY2* | rna-XM_027308549.1 | Chr1 | *-* | *Coffea eugenioides* | IId |
| Opuchr01_g0002050-1.1 | Opu_chr01 | *OpWRKY1* | rna-XM_027317611.1 | Chr6 | *-* | *Coffea eugenioides* | I |
| Opuchr02_g0007230-1.1 | Opu_chr02 | *OpWRKY6* | rna-XM_027307950.1 | Chr2 | *-* | *Coffea eugenioides* | IIc |
| Opuchr02_g0001960-1.1 | Opu_chr02 | *OpWRKY4* | rna-XM_027301826.1 | Chr2 | *-* | *Coffea eugenioides* | IIc |
| Opuchr02_g0002010-1.1 | Opu_chr02 | *OpWRKY5* | rna-XM_027306089.1 | Chr2 | *-* | *Coffea eugenioides* | IIe |
| Opuchr02_g0002010-1.1 | Opu_chr02 | *OpWRKY5* | rna-XM_027307638.1 | Chr2 | *-* | *Coffea eugenioides* | IIe |
| Opuchr02_g0069690-1.1 | Opu_chr02 | *OpWRKY7* | rna-XM_027301843.1 | Chr2 | *-* | *Coffea eugenioides* | IIb |
| Opuchr02_g0001210-1.1 | Opu_chr02 | *OpWRKY3* | rna-XM_027302058.1 | Chr2 | *-* | *Coffea eugenioides* | III |
| Opuchr02_g0001210-1.1 | Opu_chr02 | *OpWRKY3* | rna-XM_027306628.1 | Chr2 | *-* | *Coffea eugenioides* | III |
| Opuchr02_g0002010-1.1 | Opu_chr02 | *OpWRKY5* | rna-XM_027327641.1 | Chr8 | *-* | *Coffea eugenioides* | IIe |
| Opuchr02_g0001960-1.1 | Opu_chr02 | *OpWRKY4* | rna-XM_027326688.1 | Chr8 | *-* | *Coffea eugenioides* | IIc |
| Opuchr02_g0001210-1.1 | Opu_chr02 | *OpWRKY3* | rna-XM_027325113.1 | Chr8 | *-* | *Coffea eugenioides* | III |
| Opuchr03_g0000900-1.1 | Opu_chr03 | *OpWRKY8* | rna-XM_027311055.1 | Chr3 | *-* | *Coffea eugenioides* | IIc |
| Opuchr04_g0061040-1.1 | Opu_chr04 | *OpWRKY10* | rna-XM_027296855.1 | Chr11 | *-* | *Coffea eugenioides* | IIb |
| Opuchr04_g0006520-1.1 | Opu_chr04 | *OpWRKY9* | rna-XM_027311634.1 | Chr4 | *-* | *Coffea eugenioides* | IIa |
| Opuchr04_g0061040-1.1 | Opu_chr04 | *OpWRKY10* | rna-XM_027312997.1 | Chr4 | *-* | *Coffea eugenioides* | IIb |
| Opuchr05_g0001510-1.1 | Opu_chr05 | *OpWRKY11* | rna-XM_027297103.1 | Chr11 | *-* | *Coffea eugenioides* | IIe |
| Opuchr05_g0001510-1.1 | Opu_chr05 | *OpWRKY11* | rna-XM_027306867.1 | Chr2 | *-* | *Coffea eugenioides* | IIe |
| Opuchr05_g0065400-1.1 | Opu_chr05 | *OpWRKY15* | rna-XM_027314218.1 | Chr5 | *-* | *Coffea eugenioides* | I |
| Opuchr05_g0067290-1.1 | Opu_chr05 | *OpWRKY16* | rna-XM_027315040.1 | Chr5 | *-* | *Coffea eugenioides* | I |
| Opuchr05_g0010260-1.1 | Opu_chr05 | *OpWRKY12* | rna-XM_027314136.1 | Chr5 | *-* | *Coffea eugenioides* | IIb |
| Opuchr06_g0119930-1.1 | Opu_chr06 | *OpWRKY20* | rna-XM_027319779.1 | Chr1 | *-* | *Coffea eugenioides* | I |
| Opuchr06_g0119930-1.1 | Opu_chr06 | *OpWRKY20* | rna-XM_027317611.1 | Chr6 | *-* | *Coffea eugenioides* | I |
| Opuchr06_g0117310-1.1 | Opu_chr06 | *OpWRKY19* | rna-XM_027317497.1 | Chr6 | *-* | *Coffea eugenioides* | IIc |
| Opuchr06_g0105040-1.1 | Opu_chr06 | *OpWRKY17* | rna-XM_027321422.1 | Chr6 | *-* | *Coffea eugenioides* | I |
| Opuchr06_g0116050-1.1 | Opu_chr06 | *OpWRKY18* | rna-XM_027319491.1 | Chr6 | *-* | *Coffea eugenioides* | I |
| Opuchr07_g0008380-1.1 | Opu_chr07 | *OpWRKY24* | rna-XM_027294107.1 | Chr10 | *-* | *Coffea eugenioides* | IId |
| Opuchr07_g0008380-1.1 | Opu_chr07 | *OpWRKY24* | rna-XM_027295784.1 | Chr11 | *-* | *Coffea eugenioides* | IId |
| Opuchr07_g0003900-1.1 | Opu_chr07 | *OpWRKY22* | rna-XM_027296855.1 | Chr11 | *-* | *Coffea eugenioides* | IIb |
| Opuchr07_g0000470-1.1 | Opu_chr07 | *OpWRKY21* | rna-XM_027296914.1 | Chr11 | *-* | *Coffea eugenioides* | IIc |
| Opuchr07_g0004690-1.1 | Opu_chr07 | *OpWRKY23* | rna-XM_027297209.1 | Chr11 | *-* | *Coffea eugenioides* | I |
| Opuchr07_g0003900-1.1 | Opu_chr07 | *OpWRKY22* | rna-XM_027296855.1 | Chr11 | *-* | *Coffea eugenioides* | IIb |
| Opuchr07_g0004690-1.1 | Opu_chr07 | *OpWRKY23* | rna-XM_027298841.1 | Chr11 | *-* | *Coffea eugenioides* | I |
| Opuchr07_g0003900-1.1 | Opu_chr07 | *OpWRKY22* | rna-XM_027312997.1 | Chr4 | *-* | *Coffea eugenioides* | IIb |
| Opuchr07_g0077110-1.1 | Opu_chr07 | *OpWRKY27* | rna-XM_027323932.1 | Chr7 | *-* | *Coffea eugenioides* | IIc |
| Opuchr07_g0076590-1.1 | Opu_chr07 | *OpWRKY25* | rna-XM_027323312.1 | Chr7 | *-* | *Coffea eugenioides* | IIc |
| Opuchr08_g0083550-1.1 | Opu_chr08 | *OpWRKY32* | rna-XM_027306628.1 | Chr2 | *-* | *Coffea eugenioides* | III |
| Opuchr08_g0083550-1.1 | Opu_chr08 | *OpWRKY32* | rna-XM_027302058.1 | Chr2 | *-* | *Coffea eugenioides* | III |
| Opuchr08_g0083550-1.1 | Opu_chr08 | *OpWRKY32* | rna-XM_027325113.1 | Chr8 | *-* | *Coffea eugenioides* | III |
| Opuchr08_g0002460-1.1 | Opu_chr08 | *OpWRKY28* | rna-XM_027327006.1 | Chr8 | *-* | *Coffea eugenioides* | IIc |
| Opuchr08_g0009150-1.1 | Opu_chr08 | *OpWRKY29* | rna-XM_027326890.1 | Chr8 | *-* | *Coffea eugenioides* | IIa |
| Opuchr09_g0018710-1.1 | Opu_chr09 | *OpWRKY38* | rna-XM_027297103.1 | Chr11 | *-* | *Coffea eugenioides* | IIe |
| Opuchr09_g0002440-1.1 | Opu_chr09 | *OpWRKY33* | rna-XM_027302058.1 | Chr2 | *-* | *Coffea eugenioides* | III |
| Opuchr09_g0003060-1.1 | Opu_chr09 | *OpWRKY34* | rna-XM_027307638.1 | Chr2 | *-* | *Coffea eugenioides* | IIe |
| Opuchr09_g0018710-1.1 | Opu_chr09 | *OpWRKY38* | rna-XM_027306867.1 | Chr2 | *-* | *Coffea eugenioides* | IIe |
| Opuchr09_g0007470-1.1 | Opu_chr09 | *OpWRKY35* | rna-XM_027304231.1 | Chr2 | *-* | *Coffea eugenioides* | III |
| Opuchr09_g0002440-1.1 | Opu_chr09 | *OpWRKY33* | rna-XM_027306628.1 | Chr2 | *-* | *Coffea eugenioides* | III |
| Opuchr09_g0003060-1.1 | Opu_chr09 | *OpWRKY34* | rna-XM_027327641.1 | Chr8 | *-* | *Coffea eugenioides* | IIe |
| Opuchr09_g0002440-1.1 | Opu_chr09 | *OpWRKY33* | rna-XM_027325113.1 | Chr8 | *-* | *Coffea eugenioides* | III |
| Opuchr09_g0010890-1.1 | Opu_chr09 | *OpWRKY37* | rna-XM_027328568.1 | Chr9 | *-* | *Coffea eugenioides* | IIb |
| Opuchr09_g0089700-1.1 | Opu_chr09 | *OpWRKY40* | rna-XM_027328173.1 | Chr9 | *-* | *Coffea eugenioides* | IIe |
| Opuchr09_g0099250-1.1 | Opu_chr09 | *OpWRKY41* | rna-XM_027327963.1 | Chr9 | *-* | *Coffea eugenioides* | I |
| Opuchr09_g0024990-1.1 | Opu_chr09 | *OpWRKY39* | rna-XM_027329781.1 | Chr9 | *-* | *Coffea eugenioides* | IIc |
| Opuchr10_g0007130-1.1 | Opu_chr10 | *OpWRKY42* | rna-XM_027294107.1 | Chr10 | *-* | *Coffea eugenioides* | IId |
| Opuchr10_g0010270-1.1 | Opu_chr10 | *OpWRKY43* | rna-XM_027293711.1 | Chr10 | *-* | *Coffea eugenioides* | IIc |
| Opuchr10_g0007130-1.1 | Opu_chr10 | *OpWRKY42* | rna-XM_027295784.1 | Chr11 | *-* | *Coffea eugenioides* | IId |
| Opuchr10_g0010270-1.1 | Opu_chr10 | *OpWRKY43* | rna-XM_027323312.1 | Chr7 | *-* | *Coffea eugenioides* | IIc |
| Opuchr11_g0080910-1.1 | Opu_chr11 | *OpWRKY44* | rna-XM_027295324.1 | Chr11 | *-* | *Coffea eugenioides* | IIc |
| Opuchr11_g0081300-1.1 | Opu_chr11 | *OpWRKY45* | rna-XM_027298841.1 | Chr11 | *-* | *Coffea eugenioides* | I |
| Opuchr11_g0081300-1.1 | Opu_chr11 | *OpWRKY45* | rna-XM_027297209.1 | Chr11 | *-* | *Coffea eugenioides* | I |
| Opuchr11_g0088100-1.1 | Opu_chr11 | *OpWRKY46* | rna-XM_027296914.1 | Chr11 | *-* | *Coffea eugenioides* | IIc |
| Opuchr11_g0088100-1.1 | Opu_chr11 | *OpWRKY46* | rna-XM_027298342.1 | Chr11 | *-* | *Coffea eugenioides* | IIc |

**Table S4.** Characteristics of *OpWRKYs* in *O. pumila*.

| **Gene ID** | **Gene name** | **Group** | **Conserved motif** | **Domain number** | **Zinc finger** | **Domain pattern** | **Best hit in *A. thaliana*** | **Blast e-value** | **Description for the best hit** |
| --- | --- | --- | --- | --- | --- | --- | --- | --- | --- |
| Opuchr09_g0007470-1.1 | *OpWRKY1* | III | WRKYGQK | 1 | C_2_HC | C-X_7_-C-X_23_-HXC | AT3G56400.1 | 8.00E-44 | WRKY DNA-binding protein 70 |
| Opuchr02_g0001210-1.1 | *OpWRKY2* | III | WRKYGQK | 1 | C_2_HC | C-X_7_-C-X_23_-HXC | AT2G46400.1 | 2.00E-44 | WRKY DNA-binding protein 46 |
| Opuchr09_g0002440-1.1 | *OpWRKY3* | III | WRKYGQK | 1 | C_2_HC | C-X_7_-C-X_23_-HXC | AT4G11070.1 | 1.00E-57 | WRKY family protein |
| Opuchr01_g0002050-1.1 | *OpWRKY4* | I | WRKYGQK | 2 | C_2_H_2_ | C-X_4_-C-X_22-23_-HXH | AT2G38470.1 | 1.00E-122 | WRKY DNA-binding protein 33 |
| Opuchr01_g0071450-1.1 | *OpWRKY5* | IId | WRKYGQK | 1 | C_2_H_2_ | C-X_5_-C-X_23_-HXH | AT2G30590.1 | 1.00E-125 | WRKY DNA-binding protein 21 |
| Opuchr02_g0001960-1.1 | *OpWRKY6* | IIc | WRKYGQK | 1 | C_2_H_2_ | C-X_4_-C-X_23_-HXH | AT1G64000.1 | 5.00E-57 | WRKY DNA-binding protein 56 |
| Opuchr02_g0002010-1.1 | *OpWRKY7* | IIe | WRKYGQK | 1 | C_2_H_2_ | C-X_5_-C-X_23_-HXH | AT4G01250.1 | 2.00E-79 | WRKY family protein |
| Opuchr02_g0007230-1.1 | *OpWRKY8* | IIc | WRKYGQK | 1 | C_2_H_2_ | C-X_4_-C-X_23_-HXH | AT2G44745.1 | 2.00E-38 | WRKY family protein |
| Opuchr02_g0069690-1.1 | *OpWRKY9* | IIb | WRKYGQK | 1 | C_2_H_2_ | C-X_5_-C-X_23_-HXH | AT1G68150.1 | 5.00E-80 | WRKY DNA-binding protein 9 |
| Opuchr03_g0000900-1.1 | *OpWRKY10* | IIc | WRKYGQK | 1 | C_2_H_2_ | C-X_4_-C-X_23_-HXH | AT2G47260.1 | 6.00E-55 | WRKY DNA-binding protein 23 |
| Opuchr04_g0006520-1.1 | *OpWRKY11* | IIa | WRKYGQK | 1 | C_2_H_2_ | C-X_5_-C-X_23_-HXH | AT1G80840.1 | 6.00E-99 | WRKY DNA-binding protein 40 |
| Opuchr04_g0061040-1.1 | *OpWRKY12* | IIb | WRKYGQK | 1 | C_2_H_2_ | C-X_5_-C-X_23_-HXH | AT5G15130.1 | 1.00E-90 | WRKY DNA-binding protein 72 |
| Opuchr05_g0001510-1.1 | *OpWRKY13* | IIe | WRKYGQK | 1 | C_2_H_2_ | C-X_5_-C-X_23_-HXH | AT3G58710.2 | 5.00E-65 | WRKY DNA-binding protein 69 |
| Opuchr05_g0010260-1.1 | *OpWRKY14* | IIb | WRKYGQK | 1 | C_2_H_2_ | C-X_5_-C-X_23_-HXH | AT1G62300.1 | 1.00E-145 | WRKY family protein |
| Opuchr05_g0039720-1.1 | *OpWRKY15* | I | WRKYGQK | 2 | C_2_H_2_ | C-X_4_-C-X_22-23_-HXH | AT2G04880.2 | 8.00E-79 | zinc-dependent activator protein-1 |
| Opuchr05_g0052440-1.1 | *OpWRKY16* | IIc | WRKYG**K**K | 1 | C_2_H_2_ | C-X_4_-C-X_23_-HXH | AT5G64810.1 | 4.00E-37 | WRKY DNA-binding protein 51 |
| Opuchr05_g0065400-1.1 | *OpWRKY17* | I | WRKYGQK | 2 | C_2_H_2_ | C-X_4_-C-X_22-23_-HXH | AT4G26640.2 | 1.00E-158 | WRKY family protein |
| Opuchr05_g0067290-1.1 | *OpWRKY18* | I | WRKYGQK | 2 | C_2_H_2_ | C-X_4_-C-X_22-23_-HXH | AT5G56270.1 | 0 | WRKY DNA-binding protein 2 |
| Opuchr06_g0105040-1.1 | *OpWRKY19* | I | WRKYGQK | 2 | C_2_H_2_ | C-X_4_-C-X_22-23_-HXH | AT4G30935.1 | 1.00E-125 | WRKY DNA-binding protein 32 |
| Opuchr06_g0116050-1.1 | *OpWRKY20* | I | WRKYGQK | 2 | C_2_H_2_ | C-X_4_-C-X_22-23_-HXH | AT2G37260.1 | 1.00E-104 | WRKY family protein |
| Opuchr06_g0117310-1.1 | *OpWRKY21* | IIc | WRKYGQK | 1 | C_2_H_2_ | C-X_4_-C-X_23_-HXH | AT5G43290.1 | 9.00E-54 | WRKY DNA-binding protein 49 |
| Opuchr06_g0119930-1.1 | *OpWRKY22* | I | WRKYGQK | 2 | C_2_H_2_ | C-X_4_-C-X_22-23_-HXH | AT2G38470.1 | 1.00E-123 | WRKY DNA-binding protein 33 |
| Opuchr07_g0000470-1.1 | *OpWRKY23* | IIc | WRKYGQK | 1 | C_2_H_2_ | C-X_4_-C-X_23_-HXH | AT5G13080.1 | 5.00E-52 | WRKY DNA-binding protein 75 |
| Opuchr07_g0003900-1.1 | *OpWRKY24* | IIb | WRKYGQK | 1 | C_2_H_2_ | C-X_5_-C-X_23_-HXH | AT5G15130.1 | 4.00E-99 | WRKY DNA-binding protein 72 |
| Opuchr07_g0004690-1.1 | *OpWRKY25* | I | WRKYGQK | 2 | C_2_H_2_ | C-X_4_-C-X_22-23_-HXH | AT1G13960.1 | 1.00E-137 | WRKY DNA-binding protein 4 |
| Opuchr07_g0008380-1.1 | *OpWRKY26* | IId | WRKYGQK | 1 | C_2_H_2_ | C-X_5_-C-X_23_-HXH | AT4G24240.1 | 8.00E-92 | WRKY DNA-binding protein 7 |
| Opuchr07_g0076590-1.1 | *OpWRKY27* | IIc | WRKYG**K**K | 1 | C_2_H_2_ | C-X_4_-C-X_23_-HXH | AT5G64810.1 | 1.00E-39 | WRKY DNA-binding protein 51 |
| Opuchr07_g0076660-1.1 | *OpWRKY28* | IIc | WRKYG**K**K | 1 | C_2_H_2_ | C-X_4_-C-X_23_-HXH | AT5G64810.1 | 6.00E-34 | WRKY DNA-binding protein 51 |
| Opuchr07_g0077110-1.1 | *OpWRKY29* | IIc | WRKYGQK | 1 | C_2_H_2_ | C-X_4_-C-X_23_-HXH | AT4G39410.1 | 3.00E-63 | WRKY DNA-binding protein 13 |
| Opuchr08_g0002460-1.1 | *OpWRKY30* | IIc | WRKYG**K**K | 1 | C_2_H_2_ | C-X_4_-C-X_23_-HXH | AT5G26170.1 | 1.00E-40 | WRKY DNA-binding protein 50 |
| Opuchr08_g0009150-1.1 | *OpWRKY31* | IIa | WRKYGQK | 1 | C_2_H_2_ | C-X_5_-C-X_23_-HXH | AT1G80840.1 | 6.00E-68 | WRKY DNA-binding protein 40 |
| Opuchr08_g0016990-1.1 | *OpWRKY32* | IIe | WRKYGQK | 1 | C_2_H_2_ | C-X_4_-C-X_23_-HXH | AT4G01250.1 | 5.00E-20 | WRKY family protein |
| Opuchr08_g0025550-1.1 | *OpWRKY33* | IId | WRKYGQK | 1 | C_2_H_2_ | C-X_5_-C-X_23_-HXH | AT4G31550.2 | 1.00E-119 | WRKY DNA-binding protein 11 |
| Opuchr08_g0083550-1.1 | *OpWRKY34* | III | WRKYGQK | 1 | C_2_HC | C-X_7_-C-X_23_-HXC | AT4G11070.1 | 8.00E-65 | WRKY family protein |
| Opuchr09_g0003060-1.1 | *OpWRKY35* | IIe | WRKYGQK | 1 | C_2_H_2_ | C-X_5_-C-X_23_-HXH | AT4G01250.1 | 5.00E-50 | WRKY family protein |
| Opuchr09_g0007480-1.1 | *OpWRKY36* | III | WRKYGQK | 1 | C_2_HC | C-X_7_-C-X_23_-HXC | AT2G40740.1 | 5.00E-45 | WRKY DNA-binding protein 55 |
| Opuchr09_g0010890-1.1 | *OpWRKY37* | IIb | WRKYGQK | 1 | C_2_H_2_ | C-X_5_-C-X_23_-HXH | AT1G62300.1 | 1.00E-158 | WRKY family protein |
| Opuchr09_g0018710-1.1 | *OpWRKY38* | IIe | WRKYGQK | 1 | C_2_H_2_ | C-X_5_-C-X_23_-HXH | AT1G29280.1 | 2.00E-72 | WRKY DNA-binding protein 65 |
| Opuchr09_g0024990-1.1 | *OpWRKY39* | IIc | WRKYGQK | 1 | C_2_H_2_ | C-X_4_-C-X_23_-HXH | AT4G18170.1 | 6.00E-71 | WRKY DNA-binding protein 28 |
| Opuchr09_g0089700-1.1 | *OpWRKY40* | IIe | WRKYGQK | 1 | C_2_H_2_ | C-X_5_-C-X_23_-HXH | AT2G34830.1 | 6.00E-89 | WRKY DNA-binding protein 35 |
| Opuchr09_g0099250-1.1 | *OpWRKY41* | I | WRKYGQK | 2 | C_2_H_2_ | C-X_4_-C-X_22-23_-HXH | AT5G56270.1 | 1.00E-93 | WRKY DNA-binding protein 2 |
| Opuchr10_g0007130-1.1 | *OpWRKY42* | IId | WRKYGQK | 1 | C_2_H_2_ | C-X_5_-C-X_23_-HXH | AT4G24240.1 | 1.00E-101 | WRKY DNA-binding protein 7 |
| Opuchr10_g0010270-1.1 | *OpWRKY43* | IIc | WRKYG**K**K | 1 | C_2_H_2_ | C-X_4_-C-X_23_-HXH | AT5G64810.1 | 2.00E-37 | WRKY DNA-binding protein 51 |
| Opuchr11_g0080910-1.1 | *OpWRKY44* | IIc | WRKYGQK | 1 | C_2_H_2_ | C-X_4_-C-X_23_-HXH | AT1G69310.2 | 2.00E-67 | WRKY DNA-binding protein 57 |
| Opuchr11_g0081300-1.1 | *OpWRKY45* | I | WRKYGQK | 2 | C_2_H_2_ | C-X_4_-C-X_22-23_-HXH | AT1G13960.1 | 1.00E-173 | WRKY DNA-binding protein 4 |
| Opuchr11_g0088100-1.1 | *OpWRKY46* | IIc | WRKYGQK | 1 | C_2_H_2_ | C-X_4_-C-X_23_-HXH | AT5G13080.1 | 2.00E-61 | WRKY DNA-binding protein 75 |

**Table S5.** The details of 10 motifs in the protein sequences of OpWRKYs.

| **Domain** | **E-value** | **Sites** | **Width** | **Multilevel consensus sequence** |
| --- | --- | --- | --- | --- |
| 1 | 4.6e-1010 | 46 | 31 | EVDILDDGYRWRKYGQKVVKGNPYPRSYYRC |
| 2 | 4.7e-762 | 46 | 29 | GCPVRKQVERASEDPSIVITTYEGKHNHP |
| 3 | 5.30E-242 | 10 | 40 | SDDGYNWRKYGQKQVKGSEYPRSYYKCTHPNCPVKKKVER |
| 4 | 1.80E-76 | 10 | 24 | HDGQITEIVYKGQHNHPKPQPNRR |
| 5 | 7.50E-66 | 25 | 15 | EKKVREPRVAVQTRS |
| 6 | 3.30E-40 | 7 | 41 | ELAVLKAELNRVREENKKLRELLEKVMEBYNSLQMHLMDLM |
| 7 | 8.50E-31 | 2 | 60 | IFDDGTIIPNPYPSPDCLMENYNFNYHHQGFEVSHHHHHHHFDLQVLDQQPVSFINNNFE |
| 8 | 2.80E-29 | 7 | 27 | JEQATSAJTSDPSFTAALAAAISSJIG |
| 9 | 1.30E-33 | 7 | 30 | TIPPGLSPTSLLDSPVLLSNSQAPPSPTTG |
| 10 | 9.50E-27 | 5 | 24 | LPVAATAMASTTSAAASMLLSGSS |

**Table S6.** *Cis*-acting elements analysis of *OpWRKY* genes identified in this study.

| **Gene Name** | **Motifs related to plant growth and development** | | | | | | | | | **Phytohormone responsive** | | | | | | | | | | | **Abiotic and biotic stress** | | | | |
| --- | --- | --- | --- | --- | --- | --- | --- | --- | --- | --- | --- | --- | --- | --- | --- | --- | --- | --- | --- | --- | --- | --- | --- | --- | --- |
|  | **ACE** | **box4** | **G-box** | **sp1** | **circadian** | **CCGTCC-box** | **CAT-box** | **GCN4_motif** | **TGA-element** | | **ABRE** | **ERE** | **GARE-motif** | **p-box** | **TATC-box** | **CGTCA-motif** | **TGACG-motif** | **TCA-element** | **ARE** | **TC-richrepeats** | | **MBS** | **LTR** | **WUN-motif** |  |
|  |  |  |  |  |  |  |  |  |  |  |  |  |  |  |  |  |  |  |  |  |  |  |  |  |  |
| *OpWRKY1* |  | 16 | 1 |  |  |  | 1 |  |  | | 1 | 3 |  |  |  | 2 | 2 | 1 |  |  | | 1 |  |  |  |
| *OpWRKY2* |  | 8 | 2 |  |  |  | 1 | 2 | 1 | | 3 |  |  | 1 | 1 | 5 | 5 |  | 3 |  | | 1 | 1 | 1 |  |
| *OpWRKY3* |  | 7 | 1 |  |  |  |  |  | 1 | | 1 | 3 |  | 1 |  | 5 | 5 |  | 4 | 1 | | 1 | 1 |  |  |
| *OpWRKY4* | 2 | 2 | 3 |  | 2 |  | 1 |  | 1 | | 3 | 2 | 1 |  | 1 | 4 | 4 |  | 1 | 1 | |  | 2 |  |  |
| *OpWRKY5* |  | 5 | 6 |  | 2 |  |  |  |  | | 5 | 3 |  | 1 |  | 1 | 1 |  | 3 |  | | 2 | 1 | 1 |  |
| *OpWRKY6* |  | 1 | 5 |  |  |  |  |  |  | | 5 | 3 | 1 | 1 |  | 1 | 1 | 3 | 4 |  | | 2 |  | 2 |  |
| *OpWRKY7* |  | 4 | 7 |  |  | 1 | 3 |  |  | | 4 | 3 |  | 2 |  | 3 | 3 |  | 1 |  | | 1 |  | 3 |  |
| *OpWRKY8* |  | 12 | 7 |  |  |  |  |  | 1 | | 5 | 1 |  |  |  |  |  | 1 | 3 | 1 | | 4 |  |  |  |
| *OpWRKY9* |  | 15 | 6 |  |  |  |  |  |  | | 5 | 1 | 1 | 1 |  | 3 | 3 | 2 | 1 |  | | 3 |  | 1 |  |
| *OpWRKY10* | 2 | 9 | 13 | 2 |  |  | 1 | 2 |  | | 9 | 1 |  | 1 |  | 2 | 2 | 1 | 5 |  | | 2 | 2 | 1 |  |
| *OpWRKY11* |  | 3 | 4 |  | 1 |  | 1 |  | 2 | | 4 |  |  | 1 |  | 2 | 2 | 3 | 7 | 1 | | 3 | 2 | 2 |  |
| *OpWRKY12* |  | 5 | 3 |  |  | 1 |  |  | 1 | | 2 | 3 |  |  | 1 | 5 | 5 |  | 2 |  | |  | 1 | 1 |  |
| *OpWRKY13* | 3 | 7 | 2 |  |  | 1 | 1 |  | 1 | | 2 | 4 |  |  |  |  |  | 1 | 1 | 1 | |  |  | 1 |  |
| *OpWRKY14* |  | 10 |  | 1 | 1 |  |  | 1 | 1 | |  | 1 |  |  |  | 3 | 3 |  |  | 1 | |  |  | 1 |  |
| *OpWRKY15* |  | 1 | 6 |  |  |  | 4 |  |  | | 3 |  | 1 |  |  |  |  | 1 | 2 | 1 | | 4 | 1 | 1 |  |
| *OpWRKY16* |  | 14 | 1 | 1 | 1 |  |  |  |  | | 2 | 2 |  | 1 | 3 | 1 | 1 | 4 | 1 | 4 | |  |  | 2 |  |
| *OpWRKY17* |  |  |  |  |  |  | 2 |  |  | |  | 2 |  |  |  | 1 | 1 | 3 | 7 |  | |  | 1 | 1 |  |
| *OpWRKY18* | 2 | 3 | 3 |  |  |  | 2 |  |  | | 2 |  |  | 1 |  | 1 | 1 | 2 | 2 |  | | 1 | 1 | 2 |  |
| *OpWRKY19* |  | 6 | 2 |  |  |  | 1 | 2 | 2 | | 1 | 2 |  |  |  |  |  | 1 | 2 | 3 | | 1 |  | 2 |  |
| *OpWRKY20* |  | 2 |  |  |  | 1 | 1 |  |  | |  |  |  | 1 |  | 1 | 1 | 2 | 3 |  | | 1 | 1 | 2 |  |
| *OpWRKY21* |  | 1 | 8 |  |  |  |  | 1 |  | | 6 | 1 | 2 | 2 |  |  |  | 3 | 4 |  | | 4 | 2 |  |  |
| *OpWRKY22* | 2 | 3 | 7 |  |  | 1 | 1 | 1 | 1 | | 8 | 2 |  |  | 1 | 2 | 2 | 1 | 4 |  | |  | 1 |  |  |
| *OpWRKY23* |  | 11 | 4 |  |  |  |  |  |  | | 4 | 2 | 2 |  | 1 | 1 | 1 |  | 3 | 1 | | 2 |  | 1 |  |
| *OpWRKY24* | 2 | 11 | 3 |  |  |  | 1 |  |  | | 3 |  |  |  | 1 | 5 | 5 | 1 | 2 |  | | 4 |  |  |  |
| *OpWRKY25* |  | 4 | 4 | 1 | 2 |  |  |  |  | | 3 | 3 |  |  |  | 1 | 1 |  | 3 |  | | 3 |  | 1 |  |
| *OpWRKY26* | 2 | 4 | 9 |  |  |  |  |  |  | | 7 | 1 |  |  | 1 | 3 | 3 | 1 | 2 |  | | 2 | 3 | 3 |  |
| *OpWRKY27* |  | 14 | 3 |  | 1 |  | 1 | 1 |  | | 4 | 5 |  |  |  | 2 | 2 | 3 | 1 |  | |  | 1 | 5 |  |
| *OpWRKY28* |  | 12 | 4 |  | 2 | 1 | 3 |  | 1 | | 5 | 1 | 1 |  |  | 1 | 1 |  |  |  | | 1 |  | 2 |  |
| *OpWRKY29* |  | 10 |  |  | 1 |  |  |  |  | |  |  |  | 1 |  | 1 | 1 | 1 | 2 | 1 | | 1 |  | 1 |  |
| *OpWRKY30* |  | 4 |  |  | 1 |  |  | 1 | 1 | |  |  |  | 2 | 1 | 1 | 1 | 3 | 2 |  | |  |  |  |  |
| *OpWRKY31* |  | 3 | 9 |  | 2 |  | 4 |  |  | | 8 | 1 |  |  | 1 | 2 | 2 | 1 | 1 | 3 | | 1 |  | 2 |  |
| *OpWRKY32* |  | 5 | 1 |  |  |  |  |  | 2 | | 1 | 3 |  |  | 1 | 1 | 1 | 1 | 2 | 1 | |  | 1 |  |  |
| *OpWRKY33* |  | 6 |  |  |  |  | 1 |  |  | |  | 3 |  | 1 |  | 3 | 3 | 1 | 5 |  | | 1 | 1 | 4 |  |
| *OpWRKY34* |  | 3 | 4 |  |  |  | 1 |  |  | | 2 |  |  | 1 |  | 5 | 5 |  | 2 |  | | 1 | 2 |  |  |
| *OpWRKY35* |  | 1 | 3 |  | 1 | 1 | 2 | 1 |  | | 2 |  |  | 1 | 1 | 2 | 2 |  | 5 |  | | 1 | 1 | 1 |  |
| *OpWRKY36* |  | 13 | 2 |  |  | 3 |  |  | 1 | | 2 | 2 |  |  |  |  |  | 2 | 3 | 1 | |  | 1 | 1 |  |
| *OpWRKY37* | 1 | 5 | 7 |  |  |  | 1 | 1 | 1 | | 7 |  |  | 1 |  | 6 | 6 |  | 2 |  | | 1 |  |  |  |
| *OpWRKY38* |  | 10 | 2 |  |  |  |  |  |  | | 3 | 1 |  | 1 |  | 1 | 1 |  | 7 | 1 | | 1 |  | 3 |  |
| *OpWRKY39* |  | 11 | 9 |  |  |  |  |  |  | | 7 | 1 |  |  |  | 3 | 3 |  | 5 |  | |  |  |  |  |
| *OpWRKY40* |  | 9 | 2 |  |  |  |  |  |  | | 3 | 1 |  | 1 |  | 3 | 3 | 1 | 2 |  | | 1 |  | 2 |  |
| *OpWRKY41* |  | 4 | 2 |  |  |  | 3 |  | 7 | | 2 | 6 |  |  |  |  |  | 2 |  |  | | 1 | 1 | 1 |  |
| *OpWRKY42* |  |  | 8 |  |  |  |  |  |  | | 5 | 2 | 1 | 1 | 2 | 4 | 4 | 1 | 3 | 1 | | 1 | 1 |  |  |
| *OpWRKY43* |  | 11 | 1 |  | 1 |  |  |  |  | | 1 | 2 |  | 2 |  | 1 | 1 | 1 | 1 | 1 | |  |  | 1 |  |
| *OpWRKY44* |  | 3 | 1 |  |  |  |  |  | 1 | | 1 | 2 |  | 2 |  | 3 | 3 |  | 1 |  | | 1 | 1 | 1 |  |
| *OpWRKY45* |  | 5 | 6 |  |  | 1 |  |  | 1 | | 5 | 1 |  | 1 | 1 | 2 | 2 | 1 | 3 | 1 | | 1 |  | 3 |  |
| *OpWRKY46* |  | 5 | 8 | 1 | 1 |  | 1 |  | 2 | | 7 | 7 | 1 |  |  |  |  | 1 | 5 |  | |  |  | 1 |  |

**Table S7.** Numbers and types of *WRKY* genes in higher plants.

| **Species** | **Name** | **Total** | **Group** | | | | | | | | **NG** |
| --- | --- | --- | --- | --- | --- | --- | --- | --- | --- | --- | --- |
|  |  |  | **I** | **IIa** | **IIb** | **IIc** | **IId** | **IIe** | **II** | **III** |  |
| *Sesamum indicum* | SiWRKY | 71 | 12 | 4 | 11 | 18 | 7 | 8 | 48 | 7 | 4 |
| *Cucumis sativus* | CsWRKY | 55 | 10 | 4 | 4 | 16 | 8 | 7 | 39 | 6 | 0 |
| *Arabidopsis thaliana* | AtWRKY | 72 | 13 | 4 | 7 | 18 | 7 | 9 | 45 | 14 | 0 |
| *Vitis vinifera* | VvWRKY | 59 | 12 | 3 | 8 | 15 | 7 | 6 | 39 | 6 | 2 |
| *Oryza sativa* | OsWRKY | 103 | 15 | 4 | 8 | 15 | 7 | 11 | 45 | 36 | 0 |
| *Solanum lycopersicum* | SlWRKY | 78 | 15 | 5 | 8 | 16 | 6 | 17 | 52 | 11 | 3 |
| *Linum usitatissimum* | LuWRKY | 97 | 24 | 4 | 13 | 16 | 11 | 12 | 56 | 15 | 2 |
| *Glycine max* | GmWRKY | 188 | 32 | 14 | 33 | 42 | 21 | 20 | 130 | 26 | 0 |
| *Ricinus communis* | CbWRKY | 47 | 9 | 3 | 10 | 12 | 3 | 5 | 33 | 5 | 0 |
| *Brachypodium distachyon* | BdWRKY | 86 | 15 | 3 | 6 | 21 | 6 | 10 | 46 | 23 | 2 |
| *Zea mays* | ZmWRKY | 136 | 27 | 7 | 11 | 29 | 14 | 17 | 78 | 31 | 0 |
| *Gossypium raimondii* | GrWRKY | 116 | 22 | 6 | 16 | 33 | 15 | 12 | 82 | 12 | 0 |
| *Brassica napus* | BnWRKY | 343 | 121 | 11 | 34 | 55 | 28 | 30 | 158 | 51 | 13 |
| *Hordeum vulgare* | HvWRKY | 45 | 8 | 4 | 1 | 11 | 5 | 3 | 24 | 13 | 0 |
| *Pyrus bretschneideri* | PbWRKY | 103 | 17 | 6 | 10 | 24 | 15 | 16 | 71 | 15 | 0 |
| *Actinidia spp.* | AcWRKY | 97 | 25 | 4 | 8 | 25 | 12 | 13 | 62 | 10 |  |
| *Malus domestica* | MdWRKY | 127 | 22 | 9 | 28 | 13 | 13 | 16 | 79 | 14 | 13 |
| *Hevea brasiliensis* | HbWRKY | 81 | 16 | 6 | 11 | 17 | 9 | 8 | 51 | 14 |  |
| *Arachis duranensis* | AdWRKY | 75 | 16 | 4 | 10 | 18 | 7 | 7 | 46 | 13 |  |
| *Cajanus cajan* | CcWRKY | 92 | 16 | 5 | 17 | 23 | 8 | 10 | 63 | 13 |  |
| *Cicer arietinum L.* | CaWRKY | 70 | 14 | 5 | 11 | 15 | 7 | 10 | 48 | 8 |  |
| *Lotus japonicus* | LjWRKY | 78 | 14 | 6 | 14 | 20 | 8 | 8 | 56 | 8 |  |
| *Lupinus angustifolius* | LaWRKY | 108 | 24 | 6 | 16 | 28 | 10 | 12 | 72 | 12 |  |
| *Medicago truncatula* | MtWRKY | 98 | 16 | 5 | 12 | 24 | 14 | 9 | 64 | 18 |  |
| *Phaseolus vulagaris* | PvWRKY | 88 | 14 | 5 | 15 | 25 | 7 | 10 | 62 | 12 |  |
| *Trifolium pratense* | TpWRKY | 89 | 14 | 5 | 10 | 21 | 14 | 10 | 60 | 15 |  |
| *Vigna angularis* | VaWRKY | 77 | 15 | 4 | 14 | 20 | 7 | 8 | 53 | 9 |  |
| *Vigna radiata* | VrWRKY | 76 | 16 | 1 | 18 | 15 | 7 | 7 | 48 | 12 |  |
| *Arachis ipaënsis* | AiWRKY | 77 | 14 | 4 | 10 | 18 | 7 | 9 | 48 | 15 |  |
| *O. pumila* | OpWRKY | 46 | 10 | 2 | 5 | 14 | 4 | 6 | 31 | 5 | 0 |

**Table S8** Primers used in the study.

| **Primer name** | **Squences (5' to 3')** | **Usage** |
| --- | --- | --- |
| *OpWRKY1_qF* | ACCAAGCAGGTCCAAAGGATCAAAG | qRT-PCR |
| *OpWRKY1_qR* | AACTGAGGAGACAAGAGTCGAGAGG | qRT-PCR |
| *OpWRKY2_qF* | CTAACTCAAGGTAGGGAGATGGCAAAC | qRT-PCR |
| *OpWRKY2_qR* | ACCAATGCTTCACAAGTCTCAGGAG | qRT-PCR |
| *OpWRKY3_qF* | CCACGCAGTGAAGATTCCGATAGAG | qRT-PCR |
| *OpWRKY3_qR* | CCCGTCCCTGGACAAACTTGAAC | qRT-PCR |
| *OpWRKY4_qF* | GCGGTGGTGTGGGAATGAAGAG | qRT-PCR |
| *OpWRKY4_qR* | GGCAGAGAAGGTGGAGGAATTGAC | qRT-PCR |
| *OpWRKY5_qF* | CCATCAACAGCAGCAGCAACAAC | qRT-PCR |
| *OpWRKY5_qR* | GCTATTGGTGCGTCGATACATCATTTC | qRT-PCR |
| *OpWRKY6_qF* | CGGTGCCTAAAGTGGTGTTCC | qRT-PCR |
| *OpWRKY6_qR* | GCTCCTGGGATACTTGCTGTTC | qRT-PCR |
| *OpWRKY7_qF* | TTCCCAAATCATCACCGCCTCTTTC | qRT-PCR |
| *OpWRKY7_qR* | CTGCTGCTGTTGATGCTGTTGTTG | qRT-PCR |
| *OpWRKY8_qF* | AAAGGAAAGGTGAAGGTGAGGAGAAAG | qRT-PCR |
| *OpWRKY8_qR* | ACCATCATCAAGCACATCCACATCG | qRT-PCR |
| *OpWRKY9_qF* | ACCTACGAAGGAACGCACAATCATC | qRT-PCR |
| *OpWRKY9_qR* | ATGAAGGATGCTGCTGCCGATG | qRT-PCR |
| *OpWRKY10_qF* | ATCGTCGGATGATCCAACAACAGTG | qRT-PCR |
| *OpWRKY10_qR* | GGCACAACTACACCAAGGCTTCC | qRT-PCR |
| *OpWRKY11_qF* | ACCTATGCGGACTCTGGATGATGG | qRT-PCR |
| *OpWRKY11_qR* | AGCACTTACTCGGTTCAATTCCTCTG | qRT-PCR |
| *OpWRKY12_qF* | ACAGGTTCAAAGATGTGCTGAGGAC | qRT-PCR |
| *OpWRKY12_qR* | TGGCGGAGATAGGAAGTGGATGG | qRT-PCR |
| *OpWRKY13_qF* | AAGTTGAGAGGAGCTGCCTAGACC | qRT-PCR |
| *OpWRKY13_qR* | GGGTGGTGATGGTGGTGTTTGG | qRT-PCR |
| *OpWRKY14_qF* | CCAGCACAATCATCATCAGCATCATC | qRT-PCR |
| *OpWRKY14_qR* | GAATAGTAGTCGGTGGTGAAGAGTGAG | qRT-PCR |
| *OpWRKY15_qF* | CCAAGGCGGAACCCAGAAAGTG | qRT-PCR |
| *OpWRKY15_qR* | TCGCCAGTTGTATCCATCATCCAATG | qRT-PCR |
| *OpWRKY16_qF* | CACCGTCAGAGCCAGGCATTATTAC | qRT-PCR |
| *OpWRKY16_qR* | AACAATCAGGGGAAGGATAAGGGTTTG | qRT-PCR |
| *OpWRKY17_qF* | CTCCAGTCACGATTTCAGTCCCAAC | qRT-PCR |
| *OpWRKY17_qR* | GTAGCAGCAGCAGCATCAGAGTAG | qRT-PCR |
| *OpWRKY18_qF* | AGGCATGACAATCTTGAGGTGACATC | qRT-PCR |
| *OpWRKY18_qR* | GAAGAAGCATCTACGGCATCTCCAG | qRT-PCR |
| *OpWRKY19_qF* | TGCTGCTGCTCCTGCTTCAATG | qRT-PCR |
| *OpWRKY19_qR* | AACGTCTGACCTTGTAATTCACCTTCC | qRT-PCR |
| *OpWRKY20_qF* | ACGGTGACCAATCCAATGAATCAGG | qRT-PCR |
| *OpWRKY20_qR* | TTCCCTTGACAACTTTCTGCCCATAC | qRT-PCR |
| *OpWRKY21_qF* | TCCAGAGACCCTCATAATCACCTACG | qRT-PCR |
| *OpWRKY21_qR* | TCATGCGGTACTGCTGTTGTTGG | qRT-PCR |
| *OpWRKY22_qF* | GCAGAGAAAGGATGAGGACAGAAAGG | qRT-PCR |
| *OpWRKY22_qR* | CCGCAGAAGAATGGAACATGGATATTG | qRT-PCR |
| *OpWRKY23_qF* | GGAGTGAGGTGGACATACTTGATGATG | qRT-PCR |
| *OpWRKY23_qR* | TGGACAGTCGTTGTACTTGCTTCTTC | qRT-PCR |
| *OpWRKY24_qF* | ACAGAAGAATTACCAAGCACCCTCAC | qRT-PCR |
| *OpWRKY24_qR* | CCTCCTCCTCCACCAACCATCG | qRT-PCR |
| *OpWRKY25_qF* | AAGAAGGTTGAGCGTGGAACTGATG | qRT-PCR |
| *OpWRKY25_qR* | GGTGGAGGCAGTTGGTGGTTATG | qRT-PCR |
| *OpWRKY26_qF* | CTTCTGGTCGCTGTCACTGTTCC | qRT-PCR |
| *OpWRKY26_qR* | TTAATGGGCTTCTGTCCGTACTTTCTC | qRT-PCR |
| *OpWRKY27_qF* | GGTGTTGGATAAGGGACTTAGGATTGC | qRT-PCR |
| *OpWRKY27_qR* | TCTTGACCTTCTTCTTGCCATACTTCC | qRT-PCR |
| *OpWRKY28_qF* | GGTGTTGGATAAGGGACTTAGGATTGC | qRT-PCR |
| *OpWRKY28_qR* | TCTTGACCTTCTTCTTGCCATACTTCC | qRT-PCR |
| *OpWRKY29_qF* | AGTGAAGTGGACGTGTTGGATGATG | qRT-PCR |
| *OpWRKY29_qR* | AACGATAATAACTCCTGGGATGCTGTG | qRT-PCR |
| *OpWRKY30_qF* | TGCCCAGTGAAGAAGAGAGTTGAAAG | qRT-PCR |
| *OpWRKY30_qR* | TGGTTGTGGATGCCCTCATAAGTTG | qRT-PCR |
| *OpWRKY31_qF* | TCGAATATCCAGCCCCGGAGAAG | qRT-PCR |
| *OpWRKY31_qR* | GAAGAAGCCATCTGTTCAACCATAAGC | qRT-PCR |
| *OpWRKY32_qF* | GGCTTGGCGGAAGTATGGTCAG | qRT-PCR |
| *OpWRKY32_qR* | GGCAGGACAATTAGCAACGAAACATC | qRT-PCR |
| *OpWRKY33_qF* | ACGACAACGGCGGTTAAATCTCC | qRT-PCR |
| *OpWRKY33_qR* | TATCCTTAGAACCCACCTCACATCCC | qRT-PCR |
| *OpWRKY34_qF* | CCGAGAGATGTGTCCAAGAAGAGAAAG | qRT-PCR |
| *OpWRKY34_qR* | CCATCATCACAAGGTCCTTCCAGTC | qRT-PCR |
| *OpWRKY35_qF* | AAGTAATTCCTGCTGCTACCAAGACC | qRT-PCR |
| *OpWRKY35_qR* | AGGCTGAGACTGGAGATGACGAAG | qRT-PCR |
| *OpWRKY36_qF* | CCAACCAACCAGACTTCCTCTTCAG | qRT-PCR |
| *OpWRKY36_qR* | TGGCTCCAGTTGATGATGCTGTTC | qRT-PCR |
| *OpWRKY37_qF* | TCAACTTCCCTTCGCAAACCCTTC | qRT-PCR |
| *OpWRKY37_qR* | GCTGCTGCTGCTTCAATGTCTTG | qRT-PCR |
| *OpWRKY38_qF* | TGGAGGAAGTACGGTCAGAAGCC | qRT-PCR |
| *OpWRKY38_qR* | TTGCTGGACATCCCTTTGAACTACTAC | qRT-PCR |
| *OpWRKY39_qF* | AAGGACCATCTGACCAGAACACATTG | qRT-PCR |
| *OpWRKY39_qR* | TCTCCAGCCTCAACAACAACAACTG | qRT-PCR |
| *OpWRKY40_qF* | TTCCAACAAAGTTACAAGCCAGCATTG | qRT-PCR |
| *OpWRKY40_qR* | GGGTCTGCCTCTAATTCGCCTAAATC | qRT-PCR |
| *OpWRKY41_qF* | TGAAGTGCCAACAACCAGAACCAG | qRT-PCR |
| *OpWRKY41_qR* | AAACTGTCAGGGAAGGATTGGGATTG | qRT-PCR |
| *OpWRKY42_qF* | CCGAATCACAGCCGTCTCTAATCTATC | qRT-PCR |
| *OpWRKY42_qR* | CGTGTCCGATCCAAGAGGGAAATG | qRT-PCR |
| *OpWRKY43_qF* | CACCATCAGAGCCAGGCATTACTAC | qRT-PCR |
| *OpWRKY43_qR* | ACAATCAGGGGAAGAATATGGGTTTGG | qRT-PCR |
| *OpWRKY44_qF* | GCTGCTACCGTGACTGAGATGATG | qRT-PCR |
| *OpWRKY44_qR* | GGAGGTGGTTGTTGTAGTGGACTTC | qRT-PCR |
| *OpWRKY45_qF* | AACAGCAGTCACTCTACAGCCAATATG | qRT-PCR |
| *OpWRKY45_qR* | TCGGTTGTTGCCCATTGTTCCTG | qRT-PCR |
| *OpWRKY46_qF* | CCAGCACTCATCTTCAACAACAACATC | qRT-PCR |
| *OpWRKY46_qR* | AGCAGCAGCAGAAGAAGAAGAACTAG | qRT-PCR |
| *OpUBQ-qF* | TTTTGCTGGGAAACAGTTGGAAG | qRT-PCR |
| *OpUBQ-qR* | CAGAAACCACCACGGAGACGC | qRT-PCR |
| *OpWRKY6-KF* | ATGAATTTGAGTCCTCTTACG | Gene cloning |
| *OpWRKY6-KR* | CTAAAACCGAGTGAGAAACTGTAT | Gene cloning |
| *pHB-OpWRKY6-F* | CTCTCTCTCAAGCTTGGATCCATGAATTTGAGTCCTCTTACG | Overexpression; Subcellular localization |
| *pHB-OpWRKY6-R* | GCCCTTGCTCACCATACTAGTAAACCGAGTGAGAAACTGTAT | Overexpression; Subcellular localization |
| *pHB-R* | TGTGGCCGTTTACGTCGC | Overexpression detection |
| *rolB-F* | GCTCTTGCAGTGCTAGATTT | Overexpression detection |
| *rolB-R* | GAAGGTGCAAGCTACCTCTC | Overexpression detection |
| *OpWRKY6sgRNA-F* | GATTGAAAAGTACGGTGCCTAAAG | CRISPR/Cas9 |
| *OpWRKY6sgRNA-R* | AAACCTTTAGGCACCGTACTTTTC | CRISPR/Cas9 |
| *OpWRKY6-F_75_* | TTTTGCACAATCCCATTCT | CRISPR/Cas9 detection |
| *OpWRKY6-R_552_* | GTGATGCGAGCACCGATA | CRISPR/Cas9 detection |
| *pB42AD-OpWRKY6-F* | GATTATGCCTCTCCCGAATTCATGAATTTGAGTCCTCTTACG | Yeast one-hybrid |
| *pB42AD-OpWRKY6-R* | TGGCGAAGAAGTCCACTCGAGCTAAAACCGAGTGAGAAACTGTAT | Yeast one-hybrid |
| *pOpGES-W1-F* | AATTCAAATTTGACTTTTAAAATTTGACTTTTAAAATTTGACTTTTAC | Yeast one-hybrid |
| *pOpGES-W1-R* | TCGAGTAAAAGTCAAATTTTAAAAGTCAAATTTTAAAAGTCAAATTTG | Yeast one-hybrid |
| *pOpGES-W2-F* | AATTCAAAGTTGACTTCATAAAGTTGACTTCATAAAGTTGACTTCATC | Yeast one-hybrid |
| *pOpGES-W2-R* | TCGAGATGAAGTCAACTTTATGAAGTCAACTTTATGAAGTCAACTTTG | Yeast one-hybrid |
| *pOpTDC-W1-F* | AATTCGGCCTTGACTGAAGGGCCTTGACTGAAGGGCCTTGACTGAAGC | Yeast one-hybrid |
| *pOpTDC-W1-R* | TCGAGCTTCAGTCAAGGCCCTTCAGTCAAGGCCCTTCAGTCAAGGCCG | Yeast one-hybrid |
| *pOp7DLH-W1-F* | AATTCACCAAGTCAACACTACCAAGTCAACACTACCAAGTCAACACTC | Yeast one-hybrid |
| *pOp7DLH-W1-R* | TCGAGAGTGTTGACTTGGTAGTGTTGACTTGGTAGTGTTGACTTGGTC | Yeast one-hybrid |
| *pOp7DLH-W2-F* | AATTCACTCGGTCAAACCGACTCGGTCAAACCGACTCGGTCAAACCGC | Yeast one-hybrid |
| *pOp7DLH-W2-R* | TCGAGCGGTTTGACCGAGTCGGTTTGACCGAGTCGGTTTGACCGAGTG | Yeast one-hybrid |
| *pOp7DLH-W3-F* | AATTCGTAGTTGACCGAGCGTAGTTGACCGAGCGTAGTTGACCGAGCC | Yeast one-hybrid |
| *pOp7DLH-W3-R* | TCGAGGCTCGGTCAACTACGCTCGGTCAACTACGCTCGGTCAACTACG | Yeast one-hybrid |
| *pOp10HGO-W1-F* | AATTCTGGTGGTCAAAGGTTGGTGGTCAAAGGTTGGTGGTCAAAGGTC | Yeast one-hybrid |
| *pOp10HGO-W1-R* | TCGAGACCTTTGACCACCAACCTTTGACCACCAACCTTTGACCACCAG | Yeast one-hybrid |
| *pOp10HGO-W2-F* | AATTCGAATTTGACCGGAAGAATTTGACCGGAAGAATTTGACCGGAAC | Yeast one-hybrid |
| *pOp10HGO-W2-R* | TCGAGTTCCGGTCAAATTCTTCCGGTCAAATTCTTCCGGTCAAATTCG | Yeast one-hybrid |
| *pOp10HGO-W3-F* | AATTCTAGCAGTCAACCTTTAGCAGTCAACCTTTAGCAGTCAACCTTC | Yeast one-hybrid |
| *pOp10HGO-W3-R* | TCGAGAAGGTTGACTGCTAAAGGTTGACTGCTAAAGGTTGACTGCTAG | Yeast one-hybrid |
| *pOp10HGO-W4-F* | AATTCACCTTTGACCGCCAACCTTTGACCGCCAACCTTTGACCGCCAC | Yeast one-hybrid |
| *pOp10HGO-W4-R* | TCGAGTGGCGGTCAAAGGTTGGCGGTCAAAGGTTGGCGGTCAAAGGTG | Yeast one-hybrid |
| *pOp10HGO-W5-F* | AATTCGCCCTTGACTTCTTGCCCTTGACTTCTTGCCCTTGACTTCTTC | Yeast one-hybrid |
| *pOp10HGO-W5-R* | TCGAGAAGAAGTCAAGGGCAAGAAGTCAAGGGCAAGAAGTCAAGGGCG | Yeast one-hybrid |
| *pOp7DLH-W3-mF* | AATTCGTAGAATTTTGAGCGTAGAATTTTGAGCGTAGAATTTTGAGCC | Yeast one-hybrid |
| *pOp7DLH-W3-mR* | TCGAGGCTCAAAATTCTACGCTCAAAATTCTACGCTCAAAATTCTACG | Yeast one-hybrid |
| *pOp7DLH-W2-mF* | AATTCACTCAAAATTACCGACTCAAAATTACCGACTCAAAATTACCGC | Yeast one-hybrid |
| *pOp7DLH-W2-mR* | TCGAGCGGTAATTTTGAGTCGGTAATTTTGAGTCGGTAATTTTGAGTG | Yeast one-hybrid |
| *pOp7DLH-W1-mF* | AATTCACCATAAATTCACTACCATAAATTCACTACCATAAATTCACTC | Yeast one-hybrid |
| *pOp7DLH-W1-mR* | TCGAGAGTGAATTTATGGTAGTGAATTTATGGTAGTGAATTTATGGTG | Yeast one-hybrid |
| *pOp10HGO-W1-mF* | AATTCTGGTAAAATTAGGTTGGTAAAATTAGGTTGGTAAAATTAGGTC | Yeast one-hybrid |
| *pOp10HGO-W1-mR* | TCGAGACCTAATTTTACCAACCTAATTTTACCAACCTAATTTTACCAG | Yeast one-hybrid |
| *pOp10HGO-W2-mF* | AATTCGAATAATTTTGGAAGAATAATTTTGGAAGAATAATTTTGGAAC | Yeast one-hybrid |
| *pOp10HGO-W2-mR* | TCGAGTTCCAAAATTATTCTTCCAAAATTATTCTTCCAAAATTATTCG | Yeast one-hybrid |
| *pOp10HGO-W3-mF* | AATTCTAGCTAAATTCCTTTAGCTAAATTCCTTTAGCTAAATTCCTTC | Yeast one-hybrid |
| *pOp10HGO-W3-mR* | TCGAGAAGGAATTTAGCTAAAGGAATTTAGCTAAAGGAATTTAGCTAG | Yeast one-hybrid |
| *pOp10HGO-W4-mF* | AATTCACCTAATTTTGCCAACCTAATTTTGCCAACCTAATTTTGCCAC | Yeast one-hybrid |
| *pOp10HGO-W4-mR* | TCGAGTGGCAAAATTAGGTTGGCAAAATTAGGTTGGCAAAATTAGGTG | Yeast one-hybrid |
| *pOp10HGO-W5-mF* | AATTCGCCCAATTTATCTTGCCCAATTTATCTTGCCCAATTTATCTTC | Yeast one-hybrid |
| *pOp10HGO-W5-mR* | TCGAGAAGATAAATTGGGCAAGATAAATTGGGCAAGATAAATTGGGCG | Yeast one-hybrid |
| *pOpGES-W1-mF* | AATTCAAATAATTTATTTAAAATAATTTATTTAAAATAATTTATTTAC | Yeast one-hybrid |
| *pOpGES-W1-mR* | TCGAGTAAATAAATTATTTTAAATAAATTATTTTAAATAAATTATTTG | Yeast one-hybrid |
| *pOpGES-W2-mF* | AATTCAAAGAATTTATCATAAAGAATTTATCATAAAGAATTTATCATC | Yeast one-hybrid |
| *pOpGES-W2-mR* | TCGAGATGATAAATTCTTTATGATAAATTCTTTATGATAAATTCTTTG | Yeast one-hybrid |
| *pOpTDC-W1-mF* | AATTCGGCCAATTTAGAAGGGCCAATTTAGAAGGGCCAATTTAGAAGC | Yeast one-hybrid |
| *pOpTDC-W1-mR* | TCGAGCTTCTAAATTGGCCCTTCTAAATTGGCCCTTCTAAATTGGCCG | Yeast one-hybrid |
| *0800-pOpG10H-F* | CTTGATATCGAATTCCTGCAGTTATAGCAATGAAGATTGGAATGTTTC | Dual-luciferase |
| *0800-pOpG10H-R* | CGCTCTAGAACTAGTGGATCCCGAGGTTAAATATATTCATTAACAAATACAG | Dual-luciferase |
| *0800-pOp10HGO-F* | CTTGATATCGAATTCCTGCAGTGGCAGCAGACTCATCAGCG | Dual-luciferase |
| *0800-pOp10HGO-R* | CGCTCTAGAACTAGTGGATCCGACCTGATTTTGTGATATCAAAGAAAC | Dual-luciferase |
| *0800-pOpCPR-F* | CTTGATATCGAATTCCTGCAGTCTATACATTTTATGTTATTAATCCGTATCTG | Dual-luciferase |
| *0800-pOpCPR-R* | CGCTCTAGAACTAGTGGATCCGGTTATACGGCGTCGCTGC | Dual-luciferase |
| *0800-pOpGES-F* | CTTGATATCGAATTCCTGCAGAGTTCAGTTGTTTAACGTACACTTTCG | Dual-luciferase |
| *0800-pOpGES-R* | CGCTCTAGAACTAGTGGATCCTTTTAACTCAATTTCTGAGAGACAATAAAG | Dual-luciferase |
| *0800-pOpIO-F* | CTTGATATCGAATTCCTGCAGAAGCCTTTCCTCTCATATGTTTTAATT | Dual-luciferase |
| *0800-pOpIO-R* | CGCTCTAGAACTAGTGGATCCTGTTAGCGTACGTAGCAACACTTTC | Dual-luciferase |
| *0800-pOpSLS-F* | CTTGATATCGAATTCCTGCAGAAATCCTATAATAGATATTATTGTGATTGATGT | Dual-luciferase |
| *0800-pOpSLS-R* | CGCTCTAGAACTAGTGGATCCAGAAAACTCACTAACTCACTCTCTCTCAG | Dual-luciferase |
| *0800-pOpSTR-F* | CTTGATATCGAATTCCTGCAGAGACCCTTAGGACCAAATTCTTTAAT | Dual-luciferase |
| *0800-pOpSTR-R* | CGCTCTAGAACTAGTGGATCCGGCTTCTGAACTATGCATGTGGA | Dual-luciferase |
| *0800-pOpTDC-F* | CTTGATATCGAATTCCTGCAGACTATCGGATGACTCGGAACTTAGG | Dual-luciferase |
| *0800-pOpTDC-R* | CGCTCTAGAACTAGTGGATCCTCTTTAATAAAATAAGGATGATGGAGGA | Dual-luciferase |
| *0800-pOp7DLGT-F* | CTTGATATCGAATTCCTGCAGTGCAAATGCTGAGGTCTCGC | Dual-luciferase |
| *0800-pOp7DLGT-R* | CGCTCTAGAACTAGTGGATCCTAACAACAAGTGTGATTTTTCTGAATTC | Dual-luciferase |
| *0800-pOpLAMT-F* | CTTGATATCGAATTCCTGCAGGTTAAGCCATTTTATTAACATATCATATATAGG | Dual-luciferase |
| *0800-pOpLAMT-R* | CGCTCTAGAACTAGTGGATCCGACTCTTCTCTCACTTTCTCTCTTAGTGTG | Dual-luciferase |
| *0800-pOp7DLH-F* | CTTGATATCGAATTCCTGCAGAGCAGGTCTTCAACATGGATTACA | Dual-luciferase |
| *0800-pOp7DLH-R* | CGCTCTAGAACTAGTGGATCCTTTAGAAAGAAGGAACTATGGTGAGG | Dual-luciferase |
| *0800-pOpIS-F* | CTTGATATCGAATTCCTGCAGTGCACTCATGAGATGGCAGTGT | Dual-luciferase |
| *0800-pOpIS-R* | CGCTCTAGAACTAGTGGATCCTTTCAGAGAGACGTTGATGATGGA | Dual-luciferase |
